# Lean tissue mass is associated with adverse outcomes across different stages of chronic kidney disease: a systematic review and meta-analysis

**DOI:** 10.1038/s41598-025-34111-2

**Published:** 2026-01-05

**Authors:** Matthew Tabinor, Emma Elphick, Charlotte Stephens, Nadya Wall, Azm Ul Hussain, Rafeea Shah, Michael Sagmeister, Anna M. Price, Javeria Peracha, Benjamin Anderson, Obaida Istanbuly, James H. Savage, John Belcher, Ivonne Solis-Trapala, Charles J. Ferro, Simon J. Davies

**Affiliations:** 1 Department of Renal Medicine, Royal Stoke University Hospital, University Hospitals of North Midlands NHS Trust, Stoke-on-Trent, UK; 2 Department of Renal Medicine, University Hospitals of Coventry and Warwickshire NHS Trust, Coventry, UK; 3 Department of Renal Medicine, Queen Elizabeth Hospital, University Hospitals Birmingham NHS Trust, Birmingham, UK; 4 Department of Renal Medicine, Royal Wolverhampton Hospitals NHS Trust, Wolverhampton, UK; 5 Department of Renal Medicine, Birmingham Heartlands Hospital, University Hospitals Birmingham NHS Trust, Birmingham, UK; 6 Department of Renal Medicine, Dudley Group NHS Trust, Dudley, UK; 7 School of Medicine, Keele University, Newcastle-under-Lyme, UK; 8 Department of Applied Health Sciences, College of Medicine and Health, University of Birmingham, Birmingham, UK; 9 Institute of Cardiovascular Sciences, University of Birmingham, Birmingham, UK

**Keywords:** Body composition, Bioimpedance, Chronic kidney disease, Dialysis, Kidney transplant, Lean tissue mass, Chronic kidney disease, Skeletal muscle, Prognostic markers

## Abstract

In chronic kidney disease it is hypothesised that the association between loss of lean tissue mass (LLTM) and mortality is purely a function of multimorbidity. We conducted a systematic review in CKD patients to quantify the strength of association between LLTM and mortality or frailty surrogates, including hospitalisation and quality of life (QoL). Muscle mass was estimated using different whole-body bioimpedance methods (BI-MM). Searches of electronic databases identified 132 studies for inclusion (147542 dialysis patients; 15378 CKD_G3−5_ patients; 356 kidney transplant recipients [KTR], with 14429 deaths). From 67 studies reporting unadjusted analyses, 52 (78%) demonstrated associations between LLTM and mortality. In 80 studies reporting analyses adjusting for age, sex, and multimorbidity, 59 (74% overall: 74, 67 and 100% in dialysis, CKD, and KTR studies respectively) reported an association. Meta-analysis of dialysis studies reporting adjusted survival analyses found each degree decrease in phase angle or a lean tissue index < 10th percentile was associated with a 92 and 49% higher hazard of mortality respectively. In studies reporting hospitalisation and QoL measures, 63 and 76% reported associations with BI-MM respectively. In conclusion, having accounted for multimorbidity, LLTM remained associated with mortality and frailty surrogates in CKD, irrespective of the BI-MM method used.

**Supplementary Information:**

The online version contains supplementary material available at 10.1038/s41598-025-34111-2.

## Introduction

Loss of lean tissue mass (LLTM) is common in many long-term conditions (LTCs)^1^. In chronic kidney disease (CKD), LLTM is postulated to reflect disease severity and is associated with fatigue, poor quality of life and frailty^1^. LLTM is part of the wider process of protein energy wasting (PEW) – the loss of protein and/or energy stores in CKD, which is associated with adverse outcomes, particularly as kidney function deteriorates. The pathogenesis of PEW is complex, with factors such as uraemic toxin accumulation, metabolic acidosis and breathlessness, collectively contributing to muscle deconditioning, and therefore, poorer mobility^2^. Additionally, CKD is characterised by multimorbidity and systemic inflammation, which both further accelerate LLTM^3^. However, it remains unclear whether LLTM is directly associated with adverse outcomes in CKD after controlling for the effects of multimorbidity, systemic inflammation and progressive kidney dysfunction, and whether similar associations exist in other conditions with comparable multimorbidity, such as heart failure (HF).

In CKD, LLTM commonly co-exists with tissue overhydration. Bioimpedance (BI) – a cheap, non-invasive technique which passes small alternating currents through the body, is widely used to quantify changes in body composition in CKD, by differentiating fat mass from fat free mass^4^. Single-frequency BI methods, such as phase angle (PA), derive from tissue resistance and reactance. Therefore, reduced PA, which is associated with increased mortality in CKD^5^, reflects increases in body hydration and LLTM. In contrast, multi-frequency BI methods, such as bio-impedance defined lean tissue mass (LTM), estimate body hydration and muscle mass separately, allowing for more direct assessments of the association between LLTM and adverse outcomes. Given the observed interrelationships between worsening kidney function, multimorbidity and tissue overhydration in CKD, it is crucial to determine whether the association between LLTM and adverse outcomes in CKD remains consistent as CKD progresses, particularly after adjusting for the effects of multimorbidity.

The aim of this systematic review and meta-analysis was to summarise the published evidence of the association between LLTM, using different BI methods to estimate muscle mass (BI-MM), and mortality (all-cause or cardiovascular), across different stages of CKD. By comparing analyses that did or did not adjust for comorbidities, we aimed to establish whether LLTM is associated with an increased mortality risk after controlling for multimorbidity. Where possible, we wished to quantitatively assess the strength of any associations by grouping together different BI-MM methods in meta-analyses. To further unpick the association between LLTM and mortality, we also included studies that reported frailty surrogates as secondary outcomes, including hospitalisation, and summarised these associations. Finally, we also included separate analyses for chronic heart failure - a comparator long-term condition associated with both muscle wasting and overhydration.

## Results

### Study population identified from systematic searches

**Fig. 1:**
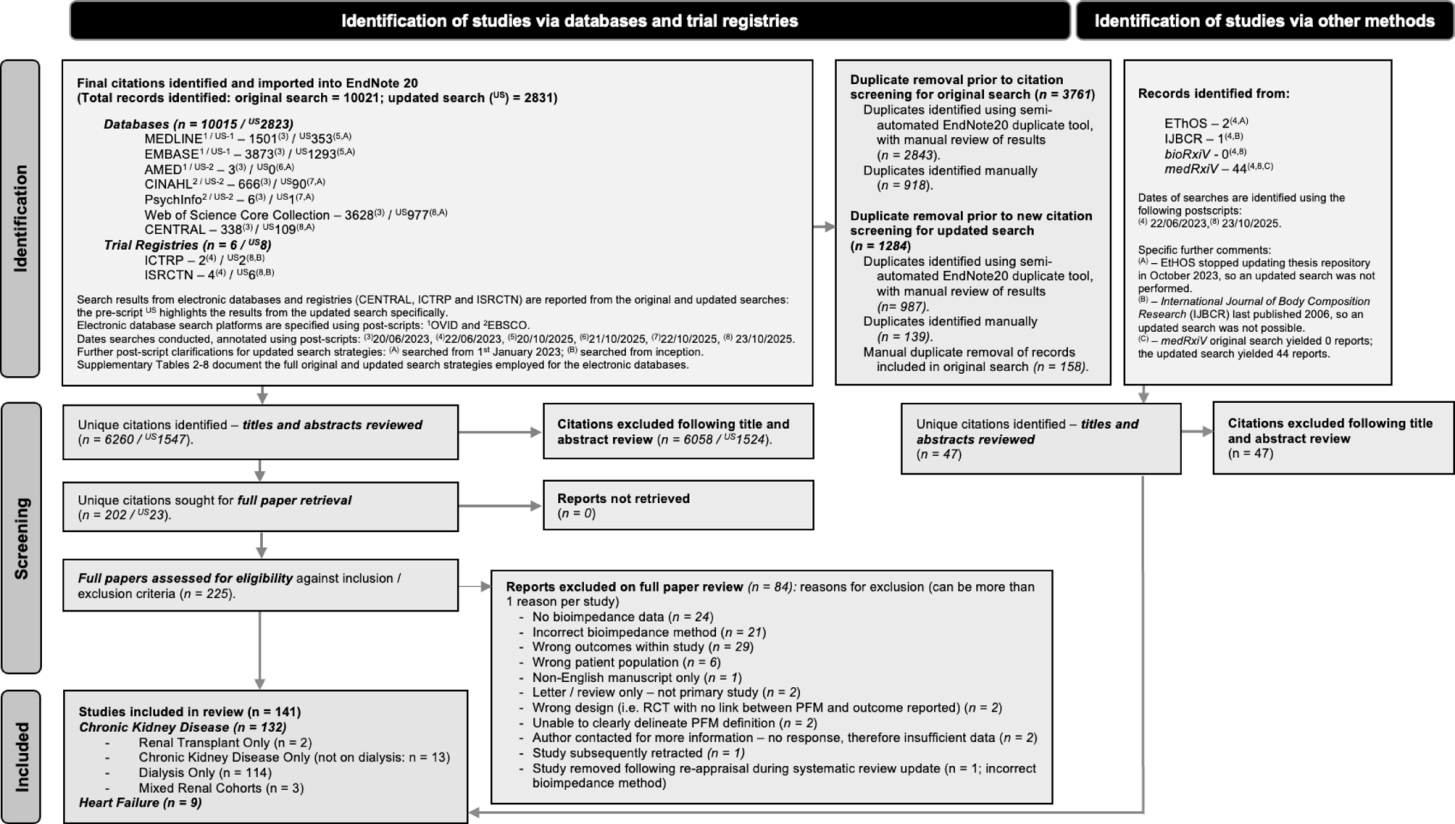
PRISMA flow diagram.

Systematic and grey literature searches yielded 12899 citations (Fig. 1), from which 7854 citations were identified following removal of duplicates. Following abstract and full paper review, 141 studies underwent data extraction: 9 in heart failure^6–14^ and 132 in kidney disease (Fig. 1). Of the kidney disease studies, 114 were in dialysis^15–128^, 13 were in CKD^129–141^, 2 were in KTR^142,143^ and 3 were in mixed kidney disease populations^144–146^. Two heart failure and 20 kidney studies were identified as describing 9 unique patient cohorts (Supplementary Table 1)^8,9,19,20,27,28,31,36,38,39,62,63,70,84,94,95,103,104,122–124^. There was considerable variation in the use of different BI measures across the studies, as summarised by (Table 1).

**Table 1:** BI-MM measures reported in studies.

BI method classification	BI method (and incremental change reported)	Studies reporting this method	BI Machines and frequency used to estimate BI measurement (SF = single frequency, MF = multi-frequency)	Timing of BI measurement	References
PA	PA (°) – per 1-degree change	26	Bioscan (*n* = 1; SF = 1) Biodynamics / Tetrapolar 450 (*n* = 3; SF = 3) InBodyS10 (*n* = 4; SF = 2, MF = 2) InBody720 (*n* = 1; MF = 1) BIA101, Akern (*n* = 4; SF = 4) Nutriguard M (*n* = 3; MF = 3) Fresenius BCM (*n* = 2; MF = 1, SF = 1*) RLJ (*n* = 5; SF = 5) Quadscan4000 (*n* = 1; SF = 1) *Unclear (n = 2)*	Pre-HD (*n* = 1) Post-HD (*n* = 13) Inter-dialytic period (*n* = 1) Peritoneum empty (*n* = 2) Peritoneum filled (*n* = 1) Non-dialysis (*n* = 7) Unclear (*n* = 3)	^7,18,21,23–25,28,29,42,52,55,56,64,69,80,95,98,102,112,128,130–132,138,143,144^
	PA (°) – categorical reporting against numerical cut off, or expressed as percentiles	23	Bioscan (*n* = 1: SF = 1) Biodynamics450 (*n* = 1: SF = 1) RLJ (*n* = 10; SF = 10) InBodyS10 (*n* = 2; SF = 2) Fresenius BCM (*n* = 1; SF = 1) Tetrapolar BIA 450 (*n* = 1; SF = 1) BIA2000 – NUTRI 4 (*n* = 1; MF = 1) Quadscan4000 (*n* = 1; MF = 1) SECA MBCA515 (*n* = 1; MF = 1) SFB7 BioImp v1.5 (*n* = 1; MF = 1) BIA101, Akern (*n* = 1; SF = 1) *Unclear (n = 2)*	Pre-HD (*n* = 3) Post-HD (*n* = 7) Pre and post-HD (*n* = 1) Peritoneum filled (*n* = 1) Non-dialysis (*n* = 6) Unclear (*n* = 6)	^6,8,10,14,15,17,20,36,37,45,46,73,74,85,91,93,94,114,118,120,127,129,136^
LTM	LTI (LTM / height^2^) – per 1 kg/m^2^ change	21	Fresenius BCM (*n* = 19; MF = 19) Tanita BC780 (*n* = 1; MF = 1) *Unclear (n = 1)*	Pre-HD (*n* = 6) Post-HD (*n* = 3) Peritoneum filled (*n* = 4) Non-dialysis (*n* = 3) Unclear (*n* = 6)	^16,30,31,35,47,59,60,62,63,72,76,82,92,97,107,122,135,139,141,142,146^
	LTI (LTM / height^2^) – categorical reporting against a numerical cut off	19	Fresenius BCM (*n* = 19; MF = 19)	Pre-HD (*n* = 11) Post-HD (*n* = 2) Peritoneum empty (*n* = 1) Peritoneum filled (*n* = 2) Unclear (*n* = 4)	^32,38,39,48,49,58,66,67,70,71,83,88,90,101,109,111,115,117,119^
	LTM (kg) – per 1 kg change	8	Fresenius BCM (*n* = 7; MF = 7) *Unclear (n = 1)*	Pre-HD (*n* = 1) Post-HD (*n* = 1) Peritoneal full (*n* = 1) Peritoneum empty (*n* = 2) Unclear (*n* = 4)	^33,34,51,57,78,110^
	LTI as part of definition of sarcopaenia diagnosis	2	Fresenius BCM (*n* = 2; MF = 2)	Post-HD (*n* = 1) Unclear (*n* = 1)	^44,96^
	LTI – ratios including fat and lean tissue mass	2	Fresenius BCM (*n* = 2; MF = 2)	Post-HD (*n* = 1) Unclear (*n* = 1)	^65,123^
SMMI	SMMI (kg/m^2^) – per 1 kg/m^2^ change	8	Fresenius BCM (*n* = 2; MF = 2) TVI-10 BC (*n* = 1; SF = 1) BIA101, Akern (*n* = 1; SF = 1) X-Contact 356 (Jawon: *n* = 1; MF = 1) MLT-550 N (*n* = 1; MF = 1) BodyStat QuadScan 1500 (*n* = 1; SF = 1) InBodyS10 (*n* = 1; MF = 1)	Post-HD (*n* = 3) Peritoneum empty (*n* = 1) Non-dialysis (*n* = 1) Unclear (*n* = 4)	^78,99,103,106,113,125,134,145^
	SMMI (kg/m^2^) – categorical reporting against a numerical cut off	5	Tanita BC706 (*n* = 2; SF = 1, MF = 1) Biodyanmics450 (*n* = 1; SF = 1) Quadscan4000 (*n* = 1; MF = 1) Fresenius BCM (*n* = 1; MF = 1)	Post-HD (*n* = 2) Non-dialysis (*n* = 1) Unclear (*n* = 2)	^68,87,104,116,137^
	SMMI / BMI (ratio) – normalised for BMI values	1	Quadscan4000 (*n* = 1; MF = 1)	Post-HD (*n* = 1)	^108^
FFM	Proportion FFM (%) – per 1% change	4	RLJ (*n* = 1; SF = 1) Maltron BF907 (*n* = 1; MF = 1) Tanita BC418 (*n* = 1; SF = 1) Bioscan (*n* = 1; SF = 1)	Non-dialysis (*n* = 3) Pre-HD (*n* = 1) Peritoneum empty (*n* = 1)	^11,12,15,133^
	FFMI (kg/m^2^) – per 1 kg / m^2^ change	1	Quantum BIA (*n* = 1; MF = 1)	Non-dialysis (*n* = 1)	^140^
	FFM value (kg) – per 1 kg change	6	Nutriguard M (*n* = 1; MF = 1) InBodyS10 (*n* = 1; SF = 1) Tanita BC601-FS (*n* = 1; MF = 1) BIA101, Akern (*n* = 1; SF = 1) *Unclear (n = 2)*	Post-HD (*n* = 4) Non-dialysis (*n* = 1) Unclear (*n* = 1)	^10,26,75,79,86,105^
LBM	Proportion FFM (%) – per 1% change	1	Fresenius BCM (*n* = 1; MF = 1)	Pre-HD (*n* = 1)	^22^
	LBM (kg) – per 1 kg change	2	Nutriguard M (*n* = 1; MF = 1) Xitron (*n* = 1; MF = 1)	Post-HD (*n* = 1) Peritoneum empty (*n* = 1)	^27,43^
BCM	BCMI ((kg/m^2^: BCM / height^2^) – categorical reporting against a numerical cut off	2	Fresenius BCM (*n* = 2; MF = 2)	Pre-HD (*n* = 2)	^81,100^
	ECM / BCM (ratio) – categorical reporting against a numerical cut off	1	Biodynamics450 (*n* = 1; SF = 1)	Unclear (*n* = 1)	^19^
	ECM / BCM (ratio) – per 1 unit ratio change	2	RLJ (*n* = 2; SF = 2)	Post-HD (*n* = 2) Peritoneum empty (*n* = 1)	^18,89^
BIVA	Vector length (expressed as change per unit of Ohms / metre)	2	RLJ (*n* = 2; SF = 2)	Post-HD (*n* = 1) Inter-dialytic (*n* = 1)	^84,102^
	Reactance (Ohms) – categorical reporting against a numerical cut off	2	Quantum BIA (*n* = 1; SF = 1) RLJ (*n* = 1; SF = 1)	Post-HD (*n* = 2)	^37,54^
	BIVA-Cachexia - defined using reference ellipses against normal population values	3	Quadscan4000 (*n* = 3; SF = 3)	Non-dialysis (*n* = 3)	^7,9,13^
Impedance ratio	Impedance ratio (BIS method where difference in impedance values at 5 and 200 kHz is expressed as a percentage)	2	Fresenius BCM (*n* = 1; MF = 1) Quadscan4000 (*n* = 1; MF = 1	Inter-dialytic (*n* = 1) Unclear (*n* = 1)	^41,53^
Muscle mass	BIS derived muscle mass (using Kaysen formula)	2	Fresenius BCM (*n* = 1; MF = 1) SFB7 ImpediMed (*n* = 1; MF = 1)	Pre-HD (*n* = 2)	^61,77^
Intracellular Water	ICW (kg) – per 1 kg change	2	Fresenius BCM (*n* = 2; MF = 2)	Pre-HD (*n* = 1) Post-HD (*n* = 1)	^50,124^
	ICW (L/m^2^) – per 1 L/m^2^ change	1	SFB7 ImpediMed (*n* = 1; MF = 1)	Pre-HD (*n* = 1)	^40^
	Proportion ICW (%)	1	Bioscan (*n* = 1; SF = 1)	Pre-HD (*n* = 1) Peritoneum full (*n* = 1)	^15^

### Methodological quality of studies

Methodological quality within the six QUIPS domains varied considerably (Supplementary Fig. 1). High risk of bias was identified in 19, 3, 6, 8, 32 and 38% of studies in the study participation, study attrition, prognostic factor measurement, outcome measurement, study confounding and the statistical analysis domains respectively (Table 2). Sampling frame reporting, handling missing data, covariate handling (definitions, measurement and inclusion within analyses) and multiple aspects of the reporting of statistical analyses, were common methodological concerns identified across many studies.

### Characteristics reported within unique cohorts identified from kidney disease and heart failure populations

Of 120 separate kidney cohorts, 78 were exclusively HD, 17 were exclusively PD, 7 were HD and PD, 13 were exclusively CKD_G3A−5_, 2 were exclusively KTR and 3 were in mixed kidney disease populations; containing collectively 147,542 dialysis (141930 HD and 5493 PD, with 2 studies not specifying the number of participants using each dialysis modality^113,145^, 15,378 CKD_G3A−5_ and 356 KTR participants (Table 2). Cohort size ranged from 18- 95,412 participants. Within cohorts, the reported mean or median age ranged between 38-76 years, the proportion of males ranged between 41-83% and the proportion of Caucasians ranged between 4-100%. The proportion of patients in kidney disease cohorts with different long-term conditions ranged between 5-69% for diabetes mellitus, 4–79% for cardiovascular disease and 47–98% for hypertension. A single cohort reported the proportion of patients with HIV/AIDS^22^, with 6 listing it as an exclusion criterion^17,18,79,114,120,141^. Where comorbidity scores were reported, 3 used Davies^47,48,131^, 19 used Charlson Comorbidity Index^15,32,33,42,52,59,65,66,75,78,89,97,108,121,126,137–139,146^ and 3 used other classifications^27,44,57^. Using reported mean or median values, BMI ranged between 21-32 kg/m^2^, serum albumin between 34- 47 g/L and high sensitivity C-reactive protein (CRP) between 0.82-122 mg/L (not counting 1 study reporting a mean CRP of 122^120^, the range was 0.82-22.80 mg/L). In cohorts containing dialysis patients, 20% reported residual kidney function, 55% dialysis dose (with a reported mean or median Kt/V ranging between 1.14-2.51), 2% previous transplant status and 19% dialysis access. Estimated glomerular filtration rate (eGFR) was reported as a mean or median for the whole cohort in 82% of non-dialysis CKD cohorts^129–140,144,146^ and 75% of KTR cohorts^142–144^; the weighted mean eGFR being 41.61 and 54.26 ml/min/1.73m^2^, respectively.

Within 8 heart failure cohorts there were 3716 participants with chronic heart failure (Table 2). Cohort size ranged between 41-2271. Within these cohorts, the reported mean or median age ranged between 63-72 years, the proportion of males ranged between 53-100% and the proportion of Caucasians ranged between 77-100%. The proportion of patients in heart failure cohorts with different long-term conditions ranged between 20-47% for diabetes mellitus and 27–67% for cardiovascular disease. Using reported mean or median values, BMI ranged between 27-29 kg/m^2^, serum albumin between 34-36 g/L, high sensitivity C-reactive protein between 3-7 mg/L and serum BNP between 370-3300pg/ml. Transthoracic echocardiogram data was reported in 88% of cohorts. Measures of kidney function were described in only 38% of heart failure cohorts; 6.7% of patients in Bieger et al. (2023)^6^ and 53.8% of patients in Orea-Tejeda et al. (2010)^13^ had an eGFR < 60 ml/min/1.73m^2^, with “extreme kidney failure” being treated as an exclusion criterion in Sobieszek et al. (2021)^14^.

### Assessing the associations between different BI-MM measures and mortality in CKD and HF

#### Dialysis dependent kidney disease

There were 11,963 deaths in 105 cohorts containing dialysis patients (Table 2) over a maximum follow up of 1–20 years (Table 3). Of the 58 studies reporting unadjusted survival analyses in dialysis-only cohorts, 2 had inconclusive results due to inconsistencies in reporting (Table 2)^113,120^. Of the remaining 56 studies, 43 (77%) reported associations between BI-MM loss and mortality. Of 72 studies reporting adjusted survival analyses in dialysis-only cohorts, 3 had inconclusive results due to inconsistent reporting^107,113,120^ and one reported cardiovascular events (Table 3)^18^. Of the remaining 68 studies, 50 (74%) showed associations between BI-MM loss and mortality: 79% in HD studies, 59% in PD studies and 40% in HD and PD studies, respectively. Assessing the impact of differences in follow-up duration, using the mean, median or maximum follow up reported for each study, BI-MM was associated with mortality in 75% of studies where follow up was < 2 years, 71% where follow up was ≥2 but < 4 years and 69% where follow up was ≥4 years. Associations between BI-MM and mortality remained despite adjusting for variables thought to be important in the causal pathway for mortality in CKD (Table 3). Age, sex and either diabetes or a comorbidity score were adjusted for in most analyses. Serum albumin, CRP or IL-6 were adjusted for in 66%, 34%, and 10% of analyses, respectively (see Table 3 for a summary of all variables adjusted for in analyses).

One study from the MONDO cohort demonstrated that LTI had different effects on survival across varying levels of inflammation and overhydration, suggesting the need to account for these factors separately when assessing associations between BI-MM loss and adverse outcomes in CKD^38^. Except for Qin et al. (2021), no study reporting two-compartment BI estimates, such as lean body mass and fat free mass, demonstrated associations with mortality. However, the estimate of effect from Qin et al. (2021) was likely imprecise, as suggested by the wide confidence intervals reported from the survival analysis (likely reflecting the small number of participants and multiple methodological concerns about the risk of bias identified from QUIPS assessments). Beberashvilli et al. (2014) demonstrated PA was no longer associated with mortality after adjusting for the Malnutrition Inflammation Atherosclerosis (MIA) score, which itself is strongly associated with LLTM. However, in this study, PA remained associated with hospitalisation and quality of life estimates.

**Table 2:**
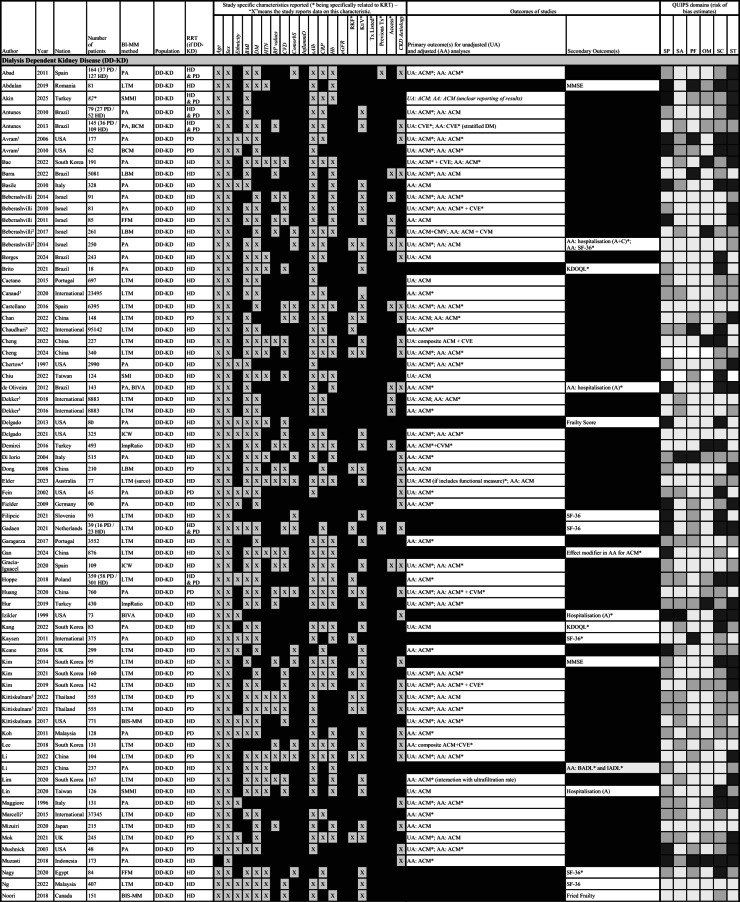

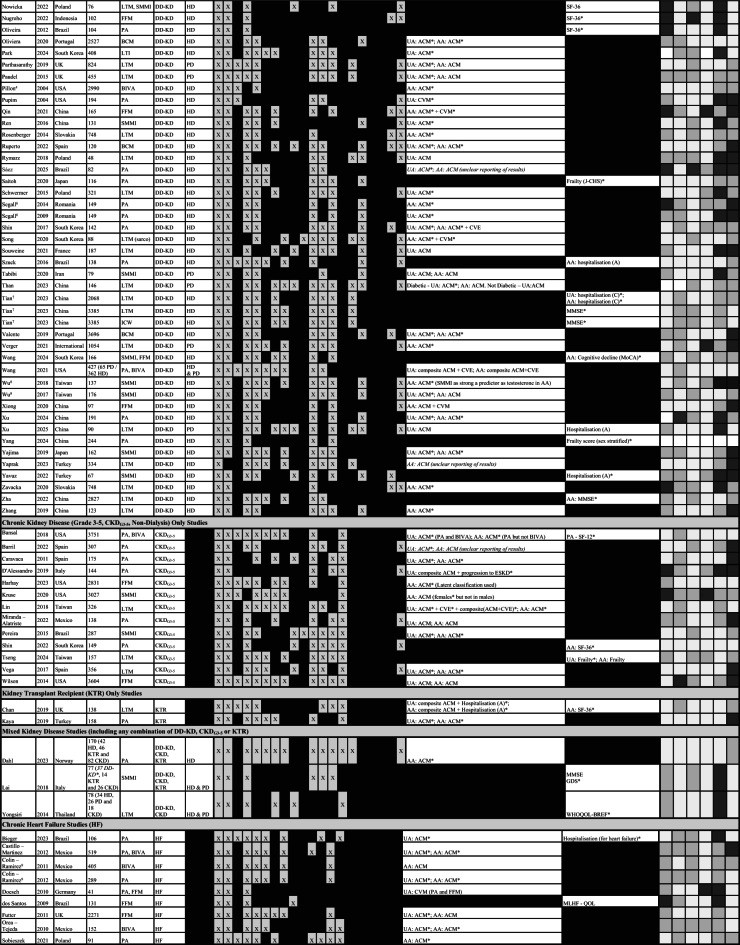
Summary of studies fulfilling inclusion criteria for review. Included studies are subdivided according to population type (DD-KD, CKD_G3−5_, KTR, mixed kidney disease and HF). Where studies are describing the same cohort of patients (described in more detail in Supplementary Table 1) they are sequentially numbered by cohort number (22 studies describe 9 separate cohorts of patients: 1 HF and 8 DD-KD). In combined HD and PD cohorts, the overall number of patients is reported, along with the number of patients on eachdialysis modality, in brackets. Where studies do not specify how many participants are on each dialysis modality, the number of patients is italicised and highlighted with a *. Primary and secondary outcomes are summarised for each study: unadjusted analyses (UA) and adjusted analyses (AA) are highlighted, along with whether they are assessing all-cause mortality (ACM), cardiovascular mortality (CVM), cardiovascular events (CVE) or a composite outcome. Statistically significant effects for the primary or secondary outcomes are highlighted with an asterisk (*). In 4 studies, the results were sufficiently unclear, due to methodological errors in reporting, that the results summaries were *italicised*. The QUIPS summaries describe the risk of bias across 6 domains: study participation (SP), study attrition (SA), prognostic factor measurement (PF), outcome measurement (OM), study confounding (SC) and statistical analysis (ST): with gradient shading representing the risk of bias – very light grey = low risk of bias, grey = uncertain risk of bias and black = high risk of bias. Abbreviations for bioimpedance defined muscle mass (BI-MM) measures include: body cell mass (BCM), bioimpedance vector analysis (BIVA), bioimpedance spectroscopy estimated muscle mass (BIS-MM), fat free mass (FFM), impedance ratio (ImpRatio), intracellular water (ICW), lean body mass (LBM), lean tissue mass (LTM), lean tissue mass used in sarcopaenia diagnosis (LTM sarco), phase angle (PA) and skeletal muscle mass index (SMMI). Abbreviations for secondary outcomes include: 12-item short survey score (SF-12), 36-item short survey score (SF-36), geriatric depression score (GDS), Japan frailty score (J-CHS), kidney disease quality of life (KDQOL), Minnesota living with heart failure questionnaire (MLHF), mini mental state examination score (MMSE), quality of life (QOL), World Health Organisation quality of life questionnaire (WHOQOL-BREF), Montreal cognitive assessment (MoCA), basic activity of daily living score (BADL), instrumental activity of daily living score (IADL). Where hospitalisation is reported in either adjusted or unadjusted analyses, this is further specified as all-cause hospitalisation (A) or cardiovascular event related hospitalisation (C). Additional abbreviations used in this table include: BMI – body mass index, ComorbS – comorbidity score, CRP – C-reactive protein, CVD – cardiovascular disease, DM – diabetes mellitus, eGFR – estimated glomerular filtration rate, Hb – haemoglobin, HTN – hypertension, InflammD – inflammatory diseases, Kt/V – dialysis dose, RKF – residual kidney function, sAlb – serum albumin and Tx – transplant.

**Table 3:**
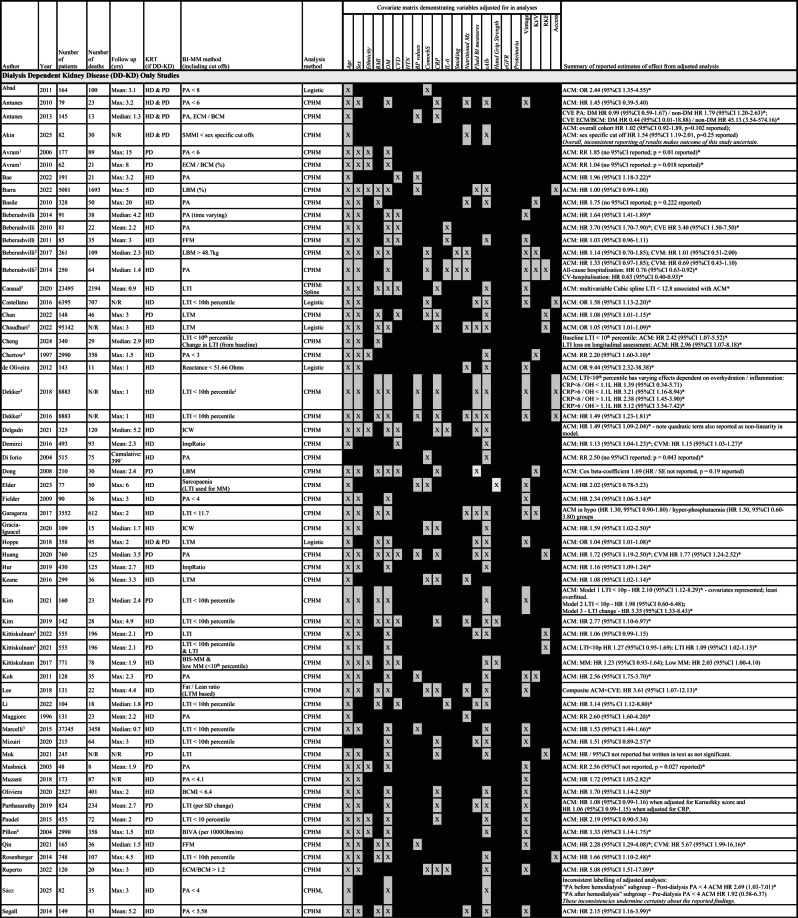

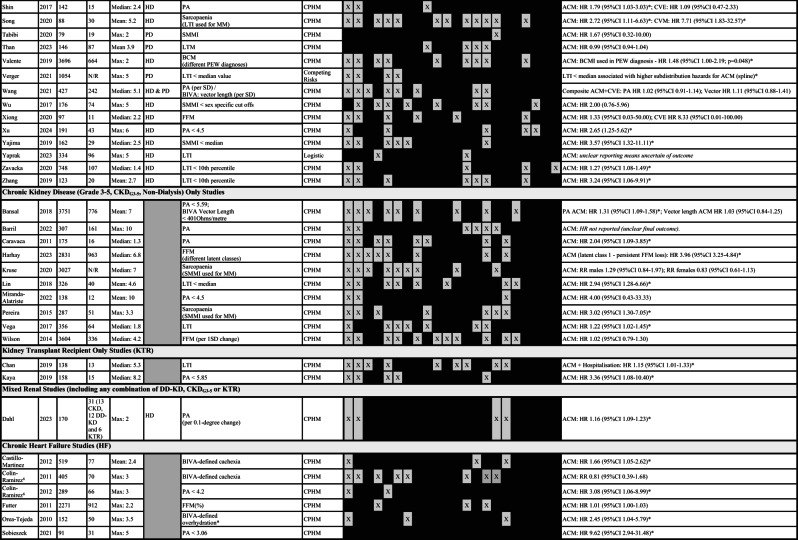
Summary of studies reporting an adjusted survival analysis for mortality. Included studies report adjusted analyses and are subdivided according to population type (D-KD, CKD_G3−5_, KTR and HF). Where studies describe the same cohort of patients (see further detail in Supplementary Table 1) they are sequentially numbered by cohort number (15 studies describe 6 separate cohorts of patients: 1 HF and 5 D-KD). Estimates of effect are summarised for each study outcome including – all-cause mortality (ACM), cardiovascular mortality (CVM), cardiovascular events (CVE) or composite outcomes. If the estimate of effect is statistically significant then this is highlighted with as an asterisk (*). One study reported an implausible follow up period (designated ^ in the table). Covariates included within the adjusted analysis are marked a “X” in the covariate matrix. Abbreviations for bioimpedance defined muscle mass (BI-MM) measures include: body cell mass (BCM), bioimpedance vector analysis (BIVA), bioimpedance spectroscopy estimated muscle mass (BIS-MM), fat free mass (FFM), impedance ratio (ImpRatio), intracellular water (ICW), lean body mass (LBM), lean tissue mass (LTM), lean tissue mass used in sarcopaenia diagnosis (LTM sarco), phase angle (PA) and skeletal muscle mass index (SMMI). Additional abbreviations used in this table include: BMI – body mass index, ComorbS – comorbidity score, CPHM – Cox proportional hazards model, CRP – C-reactive protein, CVD – cardiovascular disease, DM – diabetes mellitus, eGFR – estimated glomerular filtration rate, HD – haemodialysis, Hb – haemoglobin, HTN – hypertension, InflammD – inflammatory diseases, KRT – kidney replacement therapy, Kt/V – dialysis dose, PD – peritoneal dialysis. RKF – residual kidney function, sAlb – serum albumin and Tx – transplant.

#### CKD_G3−5_ (non dialysis)

There were 2432 deaths in 16 cohorts involving CKD_G3−5_ patients, with follow-up ranging between 1-10 years^129–140,144^. Seven of nine studies containing exclusively CKD_G3−5_ patients reporting unadjusted analyses demonstrated associations between BI-MM loss (4 out of 6 PA, 2 LTM and 1 skeletal muscle mass index) and mortality (Table 2), with two studies also showing associations with either cardiovascular events or progression to end stage kidney disease. Eleven studies containing CKD_G3−5_ patients reported adjusted analyses for mortality. One study could not be interpreted due to unclear reporting of the adjusted estimate of effect, and another was from a mixed kidney disease population, so did not report an association exclusively in CKD_G3−5_. Two studies used skeletal muscle mass index as part of the definition for sarcopaenia: Pereira et al. (2015) reported an association between sarcopaenia and mortality^137^, whereas Kruse et al. (2020) did not^134^. In the latter study, however, the number of deaths was not reported, and the nested CKD sample was small. In the remaining 7 studies reporting adjusted analyses, 5 reported associations between BI-MM loss and mortality (PA studies: 2 out of 3; FFM studies: 1 out of 2; LTM studies: 2 out of 2). Within 7 studies assessing associations between BI-MM and mortality using both adjusted and unadjusted analyses, there was complete agreement (Table 2). Age, sex and diabetes or a comorbidity score were adjusted for in most CKD_G3−5_ adjusted analyses (Table 3).

#### Kidney transplant recipients

There were 34 deaths reported in 3 cohorts involving KTR over a median follow up range of 2–8 years^142–144^. Three cohorts reported adjusted analyses containing KTR − 2 exclusively in KTR had associations between BI-MM loss and mortality, with Chan et al. (2019) additionally demonstrating an association with hospitalisation (Table 3). Furthermore, Kaya et al. (2019) reported comparable estimates of effect in both the unadjusted and adjusted survival analyses for mortality when PA < 5.85, although given the high risk of bias in the statistical methods domain of the QUIPS assessment, these results should be treated cautiously. The third adjusted analysis was in a mixed kidney disease cohort (HD, CKD and KTR; 25% of the deaths were in KTR)^144^, which demonstrated 1-degree lower PA was associated with higher mortality, albeit, this was likely an imprecise estimate due to the small number of deaths in this study.

#### Heart failure

There were 1161 deaths reported in 8 cohorts involving heart failure patients over a maximum follow up range of 2–5 years. Five out of six studies reporting unadjusted analyses demonstrated BI-MM loss was associated with higher risk of mortality (Table 2). Furthermore, when considering studies reporting adjusted analyses, four out of six studies reported BI-MM loss was associated with mortality (Table 3). When comparing the unadjusted and adjusted estimates of effect within the same studies, the association between BI-MM loss and adverse outcomes was consistent, suggesting this association was present despite adjusting for multimorbidity.

### Quantifying the association between different BI-MM methods and mortality: sub-group meta-analyses in Dialysis dependent kidney disease cohorts

**Fig. 2:**
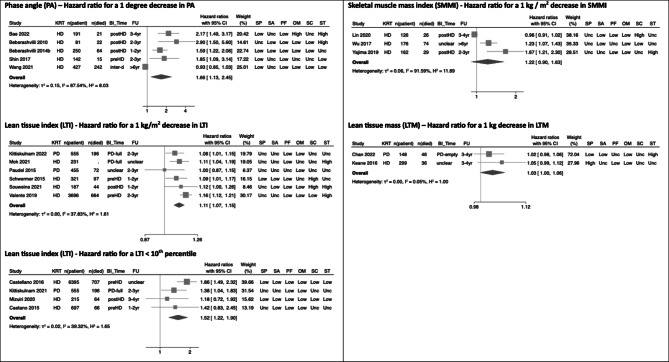
Forest plot for all BI-MM measures used in unadjusted survival analyses in DD-KD. Abbreviations used in forest plots: BI_Time – timing of BI-MM measurement; FU – follow up (maximum follow up time used for classification); HD – haemodialysis; Inter-d – interdialytic timing of BI-MM measurement; KRT – kidney replacement therapy modality; n(patient) – number of patients in study; n(died) – number of patients who died; PD – peritoneal dialysis; PreHD – pre-dialysis timing of BI-MM measurement in haemodialysis patient; PostHD – post-dialysis timing of BI-MM measurement in haemodialysis patient; PD-full – BI-MM measurement in peritoneal dialysis patient with a full peritoneum; PD-empty – BI-MM measurement in peritoneal dialysis patient with an empty peritoneum. The QUIPS domains are abbreviated as follows: SP – study participation, SA – study attrition, PF – prognostic factor measurement, OM – outcome measurement, SC – study confounding and ST – statistical analyses and reporting.

There were 20 dialysis studies reporting unadjusted analyses for all-cause mortality that were sufficiently comparable methodologically to proceed to random effects meta-analysis (Fig. 2). The pooled estimates of hazard ratios showed higher risk of mortality for a 1-degree decrease in PA (HR 1.66, 95% CI 1.13–2.45), a 1 kg/m^2^ decrease in LTI (HR 1.11, 95% CI 1.07–1.15) and a LTI < 10th percentile (HR 1.52, 95% CI 1.22–1.90). In contrast, no association was observed for a 1 kg/m^2^ decrease in skeletal muscle mass index (HR 1.22, 95% CI 0.90–1.63) and a 1 kg decrease in LTM (HR 1.03, 95%CI 1.00-1.06). In most unadjusted analyses, there were concerns regarding heterogeneity (*I*^*2*^ ranged between 0.05-92%).

**Fig. 3:**
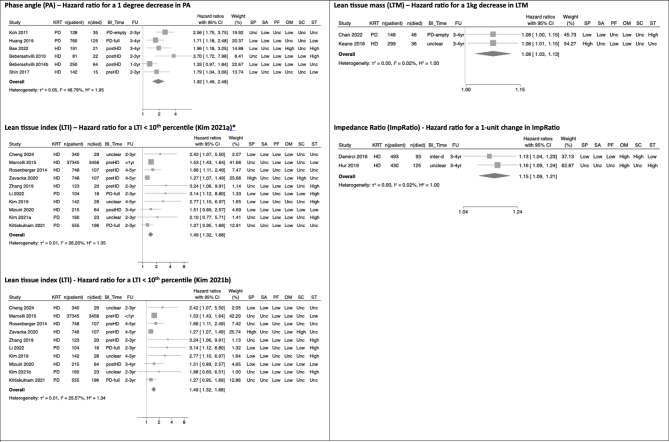
Forest plot for all BI-MM measures used in adjusted survival analyses in DD-KD. Abbreviations used in forest plots: BI_Time – timing of BI-MM measurement; FU – follow up (maximum follow up time used for classification); HD – haemodialysis; Inter-d – interdialytic timing of BI-MM measurement; KRT – kidney replacement therapy modality; n(patient) – number of patients in study; n(died) – number of patients who died; PD – peritoneal dialysis; PreHD – pre-dialysis timing of BI-MM measurement in haemodialysis patient; PostHD – post-dialysis timing of BI-MM measurement in haemodialysis patient; PD-full – BI-MM measurement in peritoneal dialysis patient with a full peritoneum; PD-empty – BI-MM measurement in peritoneal dialysis patient with an empty peritoneum. The QUIPS domains are abbreviated as follows: SP – study participation, SA – study attrition, PF – prognostic factor measurement, OM – outcome measurement, SC – study confounding and ST – statistical analyses and reporting.

There were 21 dialysis studies reporting adjusted analyses for all-cause mortality that were sufficiently comparable methodologically to proceed to random effects meta-analysis (Fig. 3). A 1 kg-decrease in LTM (HR 1.08, 95% CI 1.03–1.13) and a 1-unit increase in Impedance Ratio (HR 1.15, 95% CI 1.09–1.21) were both associated with higher risk of all-cause mortality.

**Fig. 4:**
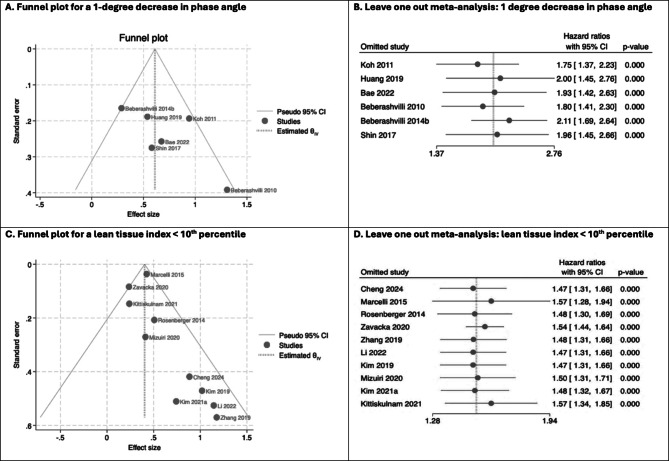
Leave one out meta-analysis and funnel plots for pooled estimates using LTI < 10th percentile and a 1-degree decrease in PA.

A 1-degree decrease in PA (HR 1.92, 95%CI 1.49–2.48) was associated with higher risk of all-cause mortality (Fig. 3). This association held in leave one out meta-analysis (Fig. 4B) and when assessed graphically, using a funnel plot, studies were distributed reasonably symmetrically (Fig. 4A), suggesting publication bias was not a major concern. The proportion of variance in the observed effects when removing the effects of sampling error (*I*^*2*^) was 49% and the between study variance of the underlying distribution of true effect size ($\:{\tau\:}^{2}$) was 0.05, suggesting there were some potential concerns about heterogeneity. One study was located on the boundary of the 95% confidence interval of the funnel plot, suggesting either small sampling variation, or potentially biased estimates^25^: from QUIPS assessments, this study was at high risk of statistical bias. Having excluded two studies at high risk of statistical bias using the QUIPS assessment, the sensitivity pooled estimate was similar in magnitude (HR 1.78, 95%CI 1.33–2.38), but concerns about heterogeneity remained (*I*^*2*^ = 55% and $\:{\tau\:}^{2}$=0.05). Using this pooled sensitivity analysis, the 95% hazard ratio prediction interval for future survival studies using a 1-degree decrease in PA was 0.57–5.54. In the 4 studies included in the sensitivity analysis, all adjusted for age, sex and diabetes mellitus / comorbidity score. However, crucially, none of these studies adjusted for albumin (Table 3).

Two separate meta-analyses were conducted in studies using LTI < 10th percentile to account for different models reported by Kim et al. (2021)^58^. Both analyses demonstrated associations with all-cause mortality (Fig. 3), with the Kim et al. 2021a model (HR 1.49, 95%CI 1.32–1.68) being selected for subsequent sensitivity analyses (Table 3 for details). Although Marcelli et al. (2015) contributed 44% towards the final pooled estimate^70^, leave one out meta-analysis did not alter the overall association (Fig. 4D). The funnel plot (Fig. 4C) demonstrated that five smaller studies were distributed asymmetrically^58,60,66,111,115^. Although this distribution may reflect small study effects, including potentially publication bias, given their small impact on the overall weighting of the pooled estimate of effect, their influence was likely to be minimal. The proportion of variance in the observed effects when removing the effects of sampling error (*I*^*2*^) was 26% and the between study variance in the underlying distribution of the true effect size ($\:{\tau\:}^{2}$) was 0.01. Collectively, this suggested low between study heterogeneity. Sensitivity analyses that excluded studies at high risk of statistical or confounding bias (using QUIPS assessments) found the pooled estimate remained robust (HR 1.52, 95%CI 1.42–1.63), with minimal concerns about heterogeneity (*I*^2^ = 0% and$\:{\:\tau\:}^{2}$<0.01), suggesting the observed between study variance in the original pooled analysis was likely explained by confounding bias or methodological errors. Using the sensitivity pooled analysis, the 95% prediction interval for the hazard ratio for future survival studies using LTI < 10th percentile was 1.37–1.70. In this pooled sensitivity analysis, 80% of studies adjusted for diabetes mellitus, age and sex, with 60% adjusting for albumin, giving greater confidence that the pooled estimates of effect for a LTI < 10th percentile were adjusted for key variables thought to be associated with mortality.

### Associations between BI-MM loss and secondary outcomes in CKD and HF

Of the 36 CKD studies that reported secondary outcomes (see Table 2 for further details), 63% of studies reporting on hospitalisation, 76% of studies reporting on health-related quality of life measures, 71% of studies reporting on cognitive function and 60% of studies reporting on frailty, demonstrated associations with BI-MM. Three dialysis studies showed BI-MM loss was associated with hospitalisation in adjusted analyses, with Beberashvilli et al. (2014) additionally demonstrating an association with health-related quality of life, again in an adjusted analysis. Two HF studies reported on secondary outcomes (Table 2): one showed lower PA was associated with hospitalisation and one showed FFM was not associated with HRQoL measures.

## Discussion

**Fig. 5:**
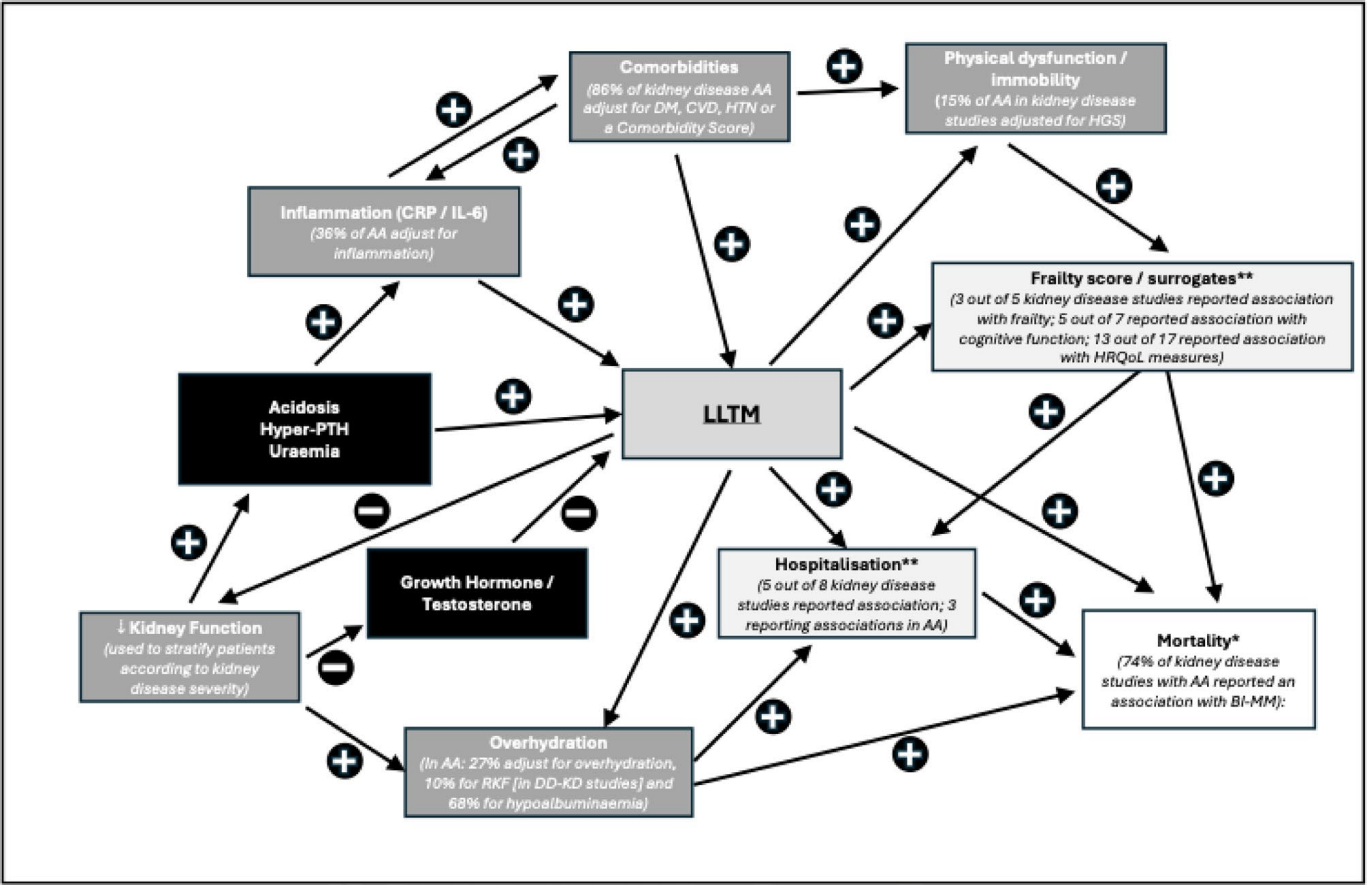
Graphical representation of the results of the systematic review. A graphical display demonstrating factors hypothesised to be important in explaining the association between LLTM and mortality in CKD, including how overhydration, although inter-related with LLTM, may explain mortality through different biological processes. Boxes highlighted with * is a primary outcome for the review and ** as a secondary outcome. AA – adjusted analysis; CKD – chronic kidney disease; CRP – C reactive protein; CVD – cardiovascular disease; DM – diabetes mellitus; HRQoL – health related quality of life measures, such as SF-36 / SF-12 and KDOQL; HTN – hypertension; IL-6 – interleukin-6; MMSE – mini mental state examination; PTH – parathyroidism; RKF – residual kidney function.

This systematic review is the first to collate comprehensive evidence using different BI-MM methods that LLTM is associated with mortality across all stages of CKD, including in kidney transplant recipients. This association remains after adjusting for potential confounders (Table 3), such as age, biological sex, multimorbidity, inflammation, serum albumin and dialysis specific factors (such as dialysis dose and residual kidney function), suggesting LLTM is important in explaining mortality in CKD. The observed associations were consistent across different BI-MM methodologies and different sub-group meta-analyses: LTI below the 10th percentile was associated with a 49% higher risk of mortality and for every degree decrease in phase angle the mortality risk rose by 92%. Furthermore, just over 70% of kidney disease studies reporting on secondary outcomes, such as hospitalisation, quality of life, cognitive function and frailty, reported associations with BI-MM. The findings of this review are summarised graphically in (Fig. 5).

A crucial study identified in this review was Dekker et al. (2018), which reported the associations between LLTM and mortality in dialysis patients for varying levels of overhydration and systemic inflammation^38^. This showed haemodialysis patients with systemic inflammation and overhydration had the strongest association between LTI and mortality, whereas those without systemic inflammation and overhydration had no association. They concluded that LLTM and overhydration are linked, and that these two processes could synergistically worsen prognosis in dialysis patients^38^. This review, which found an association between reduced PA and mortality in dialysis patients, supports this conclusion, as PA is derived from the vector that links LLTM and tissue hydration. However, our review further quantifies the LLTM-overhydration-survival relationship and found that this varies across different grades of CKD. First, we found that BI-MM loss was associated with adverse outcomes across the whole range of kidney function – including patients with kidney transplants, where the weighted mean eGFR was just over 50 ml/min/1.73m^2^. Second, despite adjustment for variables known to be directly associated with overhydration – including hypoalbuminaemia, residual kidney function (in dialysis studies) and bio-impedance defined overhydration (BI-OH), which were adjusted for in 68%, 10% and 27% of these studies (Table 3), LLTM continued to be associated with mortality in 71%, 67% and 60% respectively. Therefore, although overhydration and LLTM are inextricably linked, as suggested in extreme starvation where extracellular fluid volume does not proportionally decrease with falling muscle mass^147^, our findings suggest LLTM is associated with mortality in CKD through potentially distinct biological processes, and not directly through overhydration (Fig. 5).

LLTM has been shown to drive the metabolic phenotype of insulin resistance, along with acting as an early marker of reduced physiological reserve^148^. LLTM may contribute to adverse outcomes by increasing the risk of developing frailty, where multisystem dysregulation leads to lower physiological reserve and increased vulnerability to stressors. However, the timeframe over which LLTM is associated with adverse outcomes across different stages of CKD may differ. In our study, LLTM was associated with adverse outcomes, over shorter follow up times, in dialysis studies – especially within the MONDO cohort^31,34,38,39,70^. In contrast, LLTM was generally associated with adverse outcomes, in studies with longer periods of follow up, in both CKD_G3−5_ and kidney transplant studies, and the reported associations were weaker than in the dialysis studies. These findings support the hypothesis that there is progressive skeletal muscle dysfunction with worsening kidney disease, suggesting the need to intervene during the earlier stages of CKD, if LLTM is to become a therapeutic target to improve outcomes. Furthermore, in dialysis patients, where muscle loss is most severe, there is likely to be a limited therapeutic window where the effects of LLTM could be mitigated. This hypothesis, based on the “*catabolic crisis model*”, suggests drivers of catabolism (such as kidney disease) slow down recovery to baseline muscle function following periods of immobility / acute illness^148^. It may be that kidney failure, through uraemia, metabolic acidosis and secondary hyperparathyroidism, acts as catabolic driver, with severe kidney disease more rapidly exhausting the metabolic reserve of skeletal muscle^1,148^. This is further exacerbated by kidney failure affecting pro-anabolic hormones (Fig. 5), which act to maintain skeletal muscle health^90,149^.

These hypotheses are further supported by another finding from our systematic review– that losses in muscle function do not completely nullify the association between LLTM and adverse outcomes. Sarcopaenia, defined as LLTM and loss of muscle function (commonly measured using either hand grip strength [HGS] or gait speed), is commonly observed in ageing, and is crucial in the development of frailty^150^. Of 10 kidney disease studies adjusting for physical strength (either directly by including hand grip strength as a variable in adjusted analyses, or by using LLTM as part of the sarcopaenia definition), half reported preserved associations between LLTM and adverse outcomes. Furthermore, of the four studies expressing LLTM as sarcopaenia (including HGS in the definition), two demonstrated associations with adverse outcomes. Overall, adjustment for muscle strength / function did not completely mitigate the relationship between LLTM and adverse outcomes in CKD, suggesting losses in skeletal muscle, and not just losses of muscle function, may explain the emergence of frailty phenotypes in CKD^148^.

Our review highlights the urgent need for recommendations regarding which BI-MM measures to use in CKD and heart failure studies. The use of single frequency bioimpedance measurements, such as phase angle, influenced by both muscle mass and body hydration, does not allow for dissection of relative overhydration from fat free mass. Therefore, single frequency methods cannot accurately quantify the differential associations between LLTM and overhydration on adverse outcomes in CKD and in heart failure^151^. In contrast, BI methods based on 3 compartmental modelling, such as the Fresenius BCM device, overcome this challenge^4^ by estimating LTM separately from overhydration. Given the consistent associations across different stages of CKD between LTI (LTM indexed for height squared) and mortality, using unadjusted and adjusted analyses, and given that published reference ranges in men and women for LTI are now available^4^, we recommend the use of 3-compartment bioimpedance methods in future studies. This recommendation was similarly proposed by Mayne et al. (2023) for studies assessing BI-OH in similar populations^152^.

This review has several strengths. Firstly, this is the first review to comprehensively describe the associations between LLTM and adverse outcomes across all CKD stages and heart failure using different BI-MM measures. Secondly, our findings are in line with the results of a previous systematic review and meta-analysis which assessed the association between BI-OH and mortality in dialysis patients, with similar pooled estimates of effect being observed for a 1-degree decrease in phase angle^5^. However, given our review employed a more exhaustive search strategy, additional studies were identified and added to the pooled estimate of effect for PA. Thirdly, from citation screening to data extraction, we used two independent study reviewers, minimising the risk of individual rater biases affecting the results from the review. Fourthly, we followed recommendations to use a-priori defined operationalisation criteria when using the QUIPS tool for bias assessment^153^, minimising the risk that we would systematically bias our own assessment of bias within included studies.

However, given the observational nature of studies included in this review, there were several limitations. Firstly, all described associations cannot be interpreted as causal associations. Secondly, methodological reporting varied considerably across studies, with over a third being rated as high risk of bias from confounding and errors in statistical reporting. Therefore, despite efforts to mitigate the effect of bias on our meta-analysis estimates, the results should still be interpreted with caution. Thirdly, there was widespread variation in how LLTM was defined, particularly when BI-MM was reported in survival analysis as a categorical variable, with wide variation in the cut offs used, which precluded more extensive meta-analysis. As such, future research using BI-MM should use standardised cut offs where possible, or report BI-MM as a continuous variable, when conducting survival analyses – which would yield more meaningful inter-study comparisons, and allow for more extensive meta-analyses to be conducted. Fourthly, there was considerable variation in variable inclusion across adjusted analyses, with only consistent adjustment for age, sex and diabetes / comorbidity score. As such, the possibility of residual confounding affecting the described associations, due to differential adjustment for comorbidities across studies, remains. Finally, our study was limited by our inability to adjust for the effect that LLTM has on the measurement of kidney function itself. Serum creatinine is derived from skeletal muscle, and therefore, losses of muscle mass could impact estimated GFR when used to categorise CKD. Our understanding of the associations between LLTM and mortality across different CKD stages, particularly in those with extreme LLTM, therefore may be subject to misclassification bias.

Although this review clearly establishes that LLTM is associated with mortality across all stages of CKD and in heart failure, there is no clear consensus on how BI-MM should be used in clinical practice. Given the association between LLTM and frailty (Fig. 5), and given frailty prevalence ranges between 21 and 82% in dialysis populations worldwide^154^, BI-MM could provide a non-invasive, easy to apply longitudinal assessment for changes in muscle mass in CKD, allowing identification of those most at risk of frailty. Although the bioimpedance spectroscopy to maintain renal output (BISTRO) trial failed to demonstrate any benefit from using bio-impedance defined overhydration to determine dry weight in dialysis patients, the role of BI-MM in identifying patients or targeted nutritional interventions has not yet been tested^155^. This represents a potentially novel application, since increased protein intake is recognised to reduce mortality in older adults with CKD, despite traditional concerns about accelerated disease progression. Therefore, BI-MM may help identify CKD patients at risk of frailty and potentially facilitate timely interventions, such as dietetic assessment, referral for Kidney BEAM engagement, which has been shown to reduce symptoms of fatigue in dialysis patients^156^, or exercise training, which has been shown to improve physical function in dialysis patients^157^. Furthermore, given the associations between LLTM and mortality in kidney transplant recipients, the use of BI-MM in wait-listed patients, to identify those at greatest benefit of pre-habilitation prior to transplantation, could be another important application of this technology^158^.

## Methods

### Study registration and resign

The systematic review was prospectively registered with the International Prospective Register for Systematic Reviews (PROSPERO 2021 CRD42021240688), with the design guided by the Preferred Reporting Items for Systematic Review and Meta-analysis (PRISMA)^159^.

### Search criteria

The study population was adults (≥ 18 years) with either CKD grade 3–5 (CKD_G3−5_), kidney transplant recipients (KTR), dialysis dependent kidney disease (DD-KD) or chronic stable heart failure who had at least one measurement of whole-body BI-MM. The primary outcome was all-cause mortality. Secondary outcomes included cardiovascular mortality, hospitalisation, falls, quality of life measures and cognitive function.

### Search strategy

A comprehensive electronic systematic search of MEDLINE, EMBASE, PsychINFO, Web of Science Core Collection, AMED, CINAHL and the Cochrane Register for Controlled Trials (CENTRAL) was originally conducted from inception until the 20th June 2023. A full updated search was conducted from the 1st January 2023 to the 20th -23rd October 2025 to identify additional citations prior to publication. The updated search included a six month overlap with the original search to allow for maximal capture of newly published studies, as well as to compensate for indexing delays in electronic databases. The search strategy used a combination of medical subject headings (MeSH) and free text search terms chosen a-priori for electronic database searches. Searches of grey literature sources, including pre-print databases (*bioRxiV* and *medRxiV*), electronic thesis repositories (EThOS) and trial registries (International Standard Randomised Controlled Trial Number and the WHO International Clinical Trials Registry Platforms), used a simpler combination of free text terms, as more complex searches using Boolean operators are not possible on these platforms. Additionally, the *International Journal of Body Composition Research* (IJBCR) was searched manually as this is not electronically indexed. All citations from electronic and manual searches were stored in EndNote20. The full search strategies employed for all major electronic databases are provided in Supplementary Tables 2–8.

### Study selection and data extraction

Duplicate screening was conducted using both semi-automatic (in EndNote20) and manual processes (Fig. 1). For the updated search, an additional duplicate removal step was performed to exclude citations already identified in the original search. Following duplicate removal, citations were transferred from EndNote20 to Rayyan to assist with screening^160^. Abstracts were reviewed by two independent researchers (MT all citations, with EE, AUH, RS, MS, CS, JP, BA, AMP, NW, OI or JS as the second reviewer). Studies were excluded if they reported data from acute kidney injury patients, recently hospitalised cohorts (including decompensated heart failure) or if they reported intrathoracic / segmental bioimpedance data. Citations fulfilling the inclusion criteria following abstract review then had full manuscript review by the same reviewing team, with citations being excluded if any violation of the specified exclusion criteria was found or if an English language full paper manuscript could not be sourced electronically or through external health library searches^161^. Individual papers were then extracted (MT all full papers, with EE, AUH, RS, MS, CS, BA, OI, JS, AMP or NW as second reviewer) using a standardised piloted template (on Microsoft Excel), based on the principles outlined by Riley et al. 2019 (CHARMS-PF^162^, which included assessments for the risk of bias (ROB). No a-priori assumptions were made about data quality during data extraction. If disagreements between two reviewers could not be resolved following discussion, then adjudication was sought by a third reviewer (SJD).

### Risk of bias (ROB) assessment

The Quality in Prognostic Studies (QUIPS)^153^ tool, a validated assessment method used in a previous meta-analysis of bioimpedance defined overhydration, was employed to assess for ROB^5^. QUIPS uses six separate domains (participation, attrition, prognostic factor measurement, outcome measurement, confounding and statistical analyses), with each domain containing a series of signalling items that assist the reviewer in determining the ROB for each domain. Full details of how the QUIPS tool was operationalised in this review can be found in Supplementary Table 9.

### Narrative review method

Study characteristics, demographics, whole body BI-MM methods, outcomes and methods for reporting estimates of effect were systematically described separately for both the kidney disease and heart failure studies. BI measurement method was characterised by the timing of the BI measurement, the device used (along with the frequency used to determine the reading) and the method itself: single-frequency methods including phase angle (PA), fat free mass (FFM) or bioimpedance vector analysis (BIVA), with multi-frequency methods including impedance ratio (IR), lean tissue / body mass estimation (LTM / LBM, expressed as absolute values or indexed to the square of the height) and body cell mass estimation (BCM, expressed as absolute values or indexed to the square of the height), as previously reported^5,163^. Estimates of effect, when reported using both unadjusted and adjusted analyses, were compared to assess the consistency of these estimates when adjusting for the effect of multimorbidity.

### Statistical analysis and meta-analysis

Where studies had sufficient homogeneity within study reporting, including in the reported BI-MM methods, they were considered suitable for quantitative pooling. Where more than one study reported on outcomes from the same patient cohort with the same BI-MM measure, careful consideration was given to the survival analysis method, the number of events reported and the duration of the follow up period when choosing to include the study in the meta-analysis (Supplementary Table 1). Separate analyses were performed for studies reporting unadjusted and adjusted analyses, and additionally, where time-varying analyses were identified, these were analysed separately from non-time varying analyses. For studies reporting PA as a continuous variable, the effect of PA was expressed for a 1-degree decrease in PA. Random effects meta-analysis using the restricted maximum likelihood (REML) estimation, along with the calculation of two separate statistics used to assess heterogeneity within meta-analyses (*I*^*2*^ and *Tau*^*2*^), were performed using STATA SE 16.2. Funnel plots were used to assess symmetry of distribution around the pooled estimate of effect to potentially identify publication bias. Leave one out meta-analysis was used to assess the robustness of pooled estimates when removing individual studies. Confidence and prediction intervals are reported, allowing for more informative inferences to be made from the meta-analysis^164^. Prediction intervals are a useful measure of uncertainty for meta-analyses that capture the likely effect size of a new (similar) study based on the included studies. In comparison, confidence intervals reflect the uncertainty around the point estimate, but provide an incomplete summary of the underlying heterogeneity in the meta-analysis^164^.

## Supplementary Information

Below is the link to the electronic supplementary material.

Supplementary Material 1

## Data Availability

The datasets generated for this study are available from the corresponding author on reasonable request.
