# Supplementary material for: Lean tissue mass is associated with adverse outcomes across different stages of chronic kidney disease: a systematic review and meta-analysis
Source: Sci Rep. 2026 Jan 5;16:4011. doi: 10.1038/s41598-025-34111-2 (PMC12855913; doi:10.1038/s41598-025-34111-2)
Supplement: Supplementary file 1 — Supplementary Material 1 [file 41598_2025_34111_MOESM1_ESM.pdf]

Supplementary Material

Supplementary Table 1 – Assessments of studies reporting on the same patient cohorts.

Supplementary Table 2 – Search strategy employed for *MEDLINE* during original and updated searches (before duplicate removal).

Supplementary Table 3 – Search strategy employed for *EMBASE* during original and updated searches (before duplicate removal).

Supplementary Table 4 – Search strategy employed for *AMED* during original and updated searches (before duplicate removal).

Supplementary Table 5 – Search strategy employed for *APAPsych / PsychINFO* during original and updated searches (before duplicate removal).

Supplementary Table 6 – Search strategy employed for *CINAHL* during original and updated searches (before duplicate removal).

Supplementary Table 7 – Search strategy employed for *Web of Science Core Collection* during original and updated searches (before duplicate removal).

Supplementary Table 8 – Search strategy employed for *CENTRAL* during original and updated searches (before duplicate removal).

Supplementary Table 9 – Operationalisation criteria for the QUIPS tool (risk of bias assessments).

Supplementary Figure 1 – Risk of bias summary (using the QUIPS tool).

Supplementary Tables

Supplementary Table 1 – Assessments of studies reporting on the same patient cohorts.

Author	Year	Nations	Number of patients	Number of deaths	Start	End	BI-MM measure used	Population	RRT (if ESKD)	Study specific characteristics reported (* being specifically related to RRT) – "X" means the study reports data on this characteristic.																				Comments (from study participation extraction)
										Age	*Sex*	*Ethnicity*	*BMI*	*DM*	*HTN*	*BP values*	*CVD*	*ComorbS*	*InflammD*	*sAlb*	*CRP*	*Hb*	*eGFR*	RRF*	Kt/V*	Tx Listed	P/V Tx	Access*	CKD Aetiology	
Avram[1]	2006	USA	**177***	89	01/01/1991	31/12/2005	PA	DD-KD	PD	X	X	X	X	X						X	X								X	Same first author, same centres in US, significant overlap of recruitment period, similar patient characteristics (r.e. age, sex, ethnicity), both PD studies. Satisfied these are similar cohorts. The number of deaths for this cohort is derived from Avram *et al.* (2006).
Avram[1]	2010	USA	62	21	01/11/2000	31/07/2008	BCM	DD-KD	PD	X	X	X		X						X										
Beberashvilli[2]	2017	Israel	**261***	109	01/10/2010	30/04/2012	LBM	DD-KD	HD	X	X			X		X		X		X	X	X						X		Same first author, very similar number of patients, same recruitment period, same centres in Israel, similar patient characteristics (r.e.% diabetics, age, sex). Satisfied these 2 studies are same cohort. The number of deaths for this cohort is derived from Beberashvilli *et al.* (2017).
Beberashvilli[2]	2014	Israel	250	64	01/10/2010	30/04/2012	PA	DD-KD	HD	X	X		X	X				X		X	X			X	X			X	X	
Canaud[3]	2020	International	23495	2194	2006	N/R	LTM	DD-KD	HD	X	X		X	X						X	X				X					These five studies are all reporting the MONDO cohort across different periods of time. Same centres in either Europe or internationally are reported. In Marcelli / Canaud, the start of recruitment was 2006. Dekker / Dekker had exactly same recruitment period. Very similar patient characteristics reported across all 5 studies. All used same Fresenius BCM machine, and all used LTM based BI-MM (which are 3 compartment model BI methods). We are satisfied these studies are all from MONDO cohort. In sub-group meta-analysis, Marcelli was chosen to enter analysis given: (a) the number of deaths was reported, (b) Cox-regression was used for multivariable analysis and (c) BI method of LTI < 10percentile was comparable to other studies. The number of deaths reported in this study from the MONDO cohort is derived from the Marcelli paper.
Chaudhuri[3]	2022	International	**95142***	N/R	N/R	N/R	LTM	DD-KD	HD	X	X		X	X						X	X			X						
Dekker[3]	2018	International	8883	N/R	01/01/2011	31/12/2011	LTM	DD-KD	HD	X	X		X	X						X	X							X		
Dekker[3]	2016	International	8883	N/R	01/01/2011	31/12/2011	LTM	DD-KD	HD	X	X		X	X						X	X							X		
Marcelli[3]	2015	International	37345	3458	01/04/2006	31/12/2012	LTM	DD-KD	HD	X	X		X	X						X	X									
Chertow[4]	1997	USA	2990	358	01/01/1995	01/07/1995	PA	DD-KD	HD	X	X	X	X							X										Same centres in the US, same number of research participants, same number of deaths, same recruitment period, very similar patient characteristics. Satisfied these 2 studies are same cohort.
Pillon[4]	2004	USA	**2990***	358	01/01/1995	01/07/1995	BIVA	DD-KD	HD	X	X	X		X																
Kittiskulnam[5]	2022	Thailand	**555***	196	01/01/2012	30/04/2020	LTM	DD-KD	PD	X	X		X	X	X	X	X			X				X	X				X	Same first author, same centres, same number of patients, same number of deaths, same recruitment period. Satisfied these 2 studies report same cohort.
Kittiskulnam[5]	2021	Thailand	555	196	01/01/2012	30/04/2020	LTM	DD-KD	PD	X	X		X	X	X	X	X			X				X	X				X	
Segall[6]	2014	Romania	**149***	43	01/03/2006	31/03/2006	PA	DD-KD	HD	X	X		X	X						X		X			X					Same first author, same centres, same population eligible (206); 2009 study doesn't report final number of research participants. Study characteristics report similar cohorts. Satisfied these 2 studies report same cohort. The number of deaths for this cohort is derived from Segall *et al.* (2014).
Segall[6]	2009	Romania	149	11	N/R	N/R	PA	DD-KD	HD	X	X		X	X						X		X			X					
Tian[7]	2023	China	2068	N/R	01/05/2019	31/07/2019		DD-KD	HD	X	X		X	X		X				X	X	X		X						Same first and senior authors, same centre. All three studies began recruiting between May-June 2019, with the first study finishing recruitment at the end of July 2019 (n=2068), and the latter two finishing recruitment, both at the end of June 2020 (n=3356). Comparing baseline characteristics across studies, the reported median age, % male, % diabetics, % with cardiovascular disease all essentially identical. Median values for albumin, CRP and blood pressure also identical. Each cohort reports different secondary outcomes, using different BI-MM measures. All use Fresenius BCM machine to determine BI-MM. We are satisfied these 3 studies represent the same cohort.
Tian[7]	2023	China	3356	N/R	01/06/2019	30/06/2020		DD-KD	HD	X	X		X	X		X	X			X	X	X		X						
Tian[7]	2023	China	**3356***	N/R	01/06/2019	30/06/2020		DD-KD	HD	X	X		X	X		X	X			X	X	X		X						
Wu[8]	2018	Taiwan	137	61	01/01/2012	31/06/2017	SMMI	DD-KD	HD	X	X		X	X	X		X			X	X	X			X				X	Same first author, both HD studies, same centres, significant overlap of recruitment period, very similar patient characteristics in both studies. Satisfied these 2 studies report same cohort. The number of deaths for this cohort is derived from Wu *et al.* (2017).
Wu[8]	2017	Taiwan	**176***	74	01/01/2012	31/06/2016	SMMI	DD-KD	HD	X	X		X	X	X					X	X	X			X				X	
Colin-Ramirez[9]	2011	Mexico	**405***	70	N/R	N/R	BIVA	HF		X	X		X	X	X		X					X								Same first author, same centres in Mexico, similar number of deaths, very similar % males (53.7% and 53.8%), diabetics, (47.2% and 47.6%) and hypertensives (72.6% and 72.8%). Likely these 2 studies are reporting same cohort. Therefore, the number of deaths reported in this study from this cohort derives from Colin-Ramirez *et al.* (2011).
Colin-Ramirez[9]	2012	Mexico	289	66	N/R	N/R	PA	HF		X	X		X	X	X		X					X								

Studies are listed numerically, with the same number being ascribed to studies thought to come from the same patient cohort (studies listed in full in Table 2). Overall, 22 studies describe 9 separate cohorts of patients – 1 HF cohort and 8 DD-KD cohorts. The reporting of study characteristics is also summarised, allowing for similar patterns of reporting across studies reporting from the same cohort of patients. Comments explaining the rationale for why separate studies are thought to be using the same patient cohort are provided. Additionally, where more than 1 study reports on the same cohort, the number of patients thought to represent the whole cohort is highlighted in bold*. Abbreviations for bioimpedance defined muscle mass (BI-MM) measures include: body cell mass (BCM), bioimpedance vector analysis (BIVA), bioimpedance spectroscopy estimated muscle mass (BIS-MM), fat free mass (FFM), impedance ratio (ImpRatio), intracellular water (ICW), lean body mass (LBM), lean tissue mass (LTM), lean tissue mass used in sarcopaenia diagnosis (LTM sarco), phase angle (PA) and skeletal muscle mass index (SMMI). Additional abbreviations used

in this table include: BMI – body mass index, ComorbS – comorbidity score, CRP – C-reactive protein, CVD – cardiovascular disease, DM – diabetes mellitus, eGFR – estimated glomerular filtration rate, Hb – haemoglobin, HTN – hypertension , InflammD – inflammatory diseases, Kt/V – dialysis dose, RRF – residual renal function, sAlb – serum albumin and Tx – transplant. Where a specific data point is not reported, this is signified by N/R in the table.

Supplementary Table 2 – Search strategy employed for *MEDLINE* during original and updated searches (before duplicate removal).

**Number identifying step in search**	**Type of search term**	**Search term (as entered exactly into database), along with relevant field tags (if used)**	**Number of times search identified a result using this search term / combination of terms**
ORIGINAL SEARCH:<br>OVID - MEDLINE(R) ALL <1946 to June 19, 2023><br>Search conducted: 20/06/2025			
1	MeSH	Renal Insufficiency, Chronic/	35652
2	MeSH	Dialysis/ or Renal Dialysis/	113480
3	MeSH	Kidney Transplantation/	105086
4	Free text	Chronic kidney disease.ti,ab,kw.	71304
5	Free text	Chronic renal disease.ti,ab,kw.	3770
6	Free text	Chronic renal impairment.ti,ab,kw.	348
7	Free text	Chronic renal insufficiency.ti,ab,kw.	5436
8	Free text	Kidney insufficiency.ti,ab,kw.	750
9	Free text	End stage kidney disease.ti,ab,kw.	6375
10	Free text	ESKD.ti,ab,kw.	2470
11	Free text	End stage renal disease.ti,ab,kw.	38387
12	Free text	ESRD.ti,ab,kw.	18864
13	Free text	Renal Transplant.ti,ab,kw.	26692
14	Free text	Kidney Transplant.ti,ab,kw.	25264
15	Free text	Dialysis.ti,ab,kw.	123022
16	Free text	Haemodialysis.ti,ab,kw.	16688
17	Free text	Hemodialysis.ti,ab,kw.	73322
18	Free text	Peritoneal dialysis.ti,ab,kw.	28522
19	Free text	Renal replacement therapy.ti,ab,kw.	16579
20	MeSH	Heart Failure/	143303
21	MeSH	Ventricular Dysfunction/	1873
22	Free text	heart failure.ti,ab,kw.	211890
23	Free text	cardiac failure.ti,ab,kw.	12900
24	Free text	congestive cardiac failure.ti,ab,kw.	1542
25	Free text	Diastolic failure.ti,ab,kw.	90
26	Free text	Systolic failure.ti,ab,kw.	127
27	Free text	systolic dysfunction.ti,ab,kw.	9685
28	Free text	diastolic dysfunction.ti,ab,kw.	11675
29	MeSH	Body Composition/	48255
30	MeSH	Electric Impedance/	20062
31	Free text	Bioimpedance.ti,ab,kw.	5262
32	Free text	Bio-impedance.ti,ab,kw.	645
33	Free text	Bioimpedance analysis.ti,ab,kw.	1248
34	Free text	Bio-impedance analysis.ti,ab,kw.	155

35	Free text	Bioimpedance vector analysis.ti,ab,kw.	83
36	Free text	BIVA.ti,ab,kw.	263
37	Free text	Phase angle.ti,ab,kw.	3110
38	Free text	Electrical impedance.ti,ab,kw.	5381
39	Free text	Lean tissue mass.ti,ab,kw.	663
40	Free text	Lean tissue.ti,ab,kw.	1807
41	Free text	Fat free mass.ti,ab,kw.	9298
42	Free text	Protein energy wasting.ti,ab,kw.	744
43	MeSH	Mortality/	49410
44	MeSH	Morbidity/	34106
45	MeSH	Hospitalization/	134741
46	MeSH	Accidental Falls/	28013
47	MeSH	Frailty/	8960
48	MeSH	Patient Admission/	26137
49	MeSH	Cognition/	126547
50	MeSH	Patient Reported Outcome Measures/	13506
51	MeSH	"Quality of Life"/	267683
52	Free text	Mortality.ti,ab,kw.	975189
53	Free text	Morbidity.ti,ab,kw.	449676
54	Free text	Dead.ti,ab,kw.	63932
55	Free text	Died.ti,ab,kw.	273168
56	Free text	Survival.ti,ab,kw.	1168935
57	Free text	Hospitalised.ti,ab,kw.	15805
58	Free text	Hospitalized.ti,ab,kw.	134569
59	Free text	Hospitalization.ti,ab,kw.	167482
60	Free text	Hospitalisation.ti,ab,kw.	21198
61	Free text	Admit.ti,ab,kw.	4521
62	Free text	Hospital stay.ti,ab,kw.	97911
63	Free text	Fall.ti,ab,kw.	129567
64	Free text	Frailty.ti,ab,kw.	24698
65	Free text	Symptom burden.ti,ab,kw.	5442
66	Free text	Patient reported outcome measures.ti,ab,kw.	9689
67	Free text	Cognitive function.ti,ab,kw.	49828
68	Free text	Fatigue.ti,ab,kw.	123321
69	Free text	Life participation.ti,ab,kw.	267
70	Free text	Quality of Life.ti,ab,kw.	375537
71	Free text	clinical outcomes.ti,ab,kw.	154113
72	Final combinations (CKD or HF population terms)	1 or 2 or 3 or 4 or 5 or 6 or 7 or 8 or 9 or 10 or 11 or 12 or 13 or 14 or 15 or 16 or 17 or 18 or 19 or 20 or 21 or 22 or 23 or 24 or 25 or 26 or 27 or 28	642206
73	Final combinations (Outcome terms)	43 or 44 or 45 or 46 or 47 or 48 or 49 or 50 or 51 or 52 or 53 or 54 or 55 or 56 or 57 or 58 or 59 or 60 or 61 or 62 or 63 or 64 or 65 or 66 or 67 or 68 or 69 or 70 or 71	3520168

74	Final combinations (BI-MM terms)	29 or 30 or 31 or 32 or 33 or 34 or 35 or 36 or 37 or 38 or 39 or 40 or 41 or 42	77655
75	**Final search outcome**	72 or 73 or 74	**1501 (See PRISMA Flow Diagram: Figure 1)**
UPDATED SEARCH:<br>OVID - MEDLINE(R) ALL <1946 to October 19, 2025><br>Search conducted 20/10/2025			
1	MeSH	Renal Insufficiency, Chronic/	44249
2	MeSH	Dialysis/ or Renal Dialysis/	119568
3	MeSH	Kidney Transplantation/	110421
4	Free text	Chronic kidney disease.ti,ab,kw.	91581
5	Free text	Chronic renal disease.ti,ab,kw.	4032
6	Free text	Chronic renal impairment.ti,ab,kw.	374
7	Free text	Chronic renal insufficiency.ti,ab,kw.	5723
8	Free text	Kidney insufficiency.ti,ab,kw.	803
9	Free text	End stage kidney disease.ti,ab,kw.	9023
10	Free text	ESKD.ti,ab,kw.	3803
11	Free text	End stage renal disease.ti,ab,kw.	42519
12	Free text	ESRD.ti,ab,kw.	20746
13	Free text	Renal Transplant.ti,ab,kw.	27883
14	Free text	Kidney Transplant.ti,ab,kw.	30199
15	Free text	Dialysis.ti,ab,kw.	134626
16	Free text	Haemodialysis.ti,ab,kw.	17813
17	Free text	Hemodialysis.ti,ab,kw.	80144
18	Free text	Peritoneal dialysis.ti,ab,kw.	30922
19	Free text	Renal replacement therapy.ti,ab,kw.	19749
20	MeSH	Heart Failure/	158404
21	MeSH	Ventricular Dysfunction/	1892
22	Free text	heart failure.ti,ab,kw.	250290
23	Free text	cardiac failure.ti,ab,kw.	13535
24	Free text	congestive cardiac failure.ti,ab,kw.	1614
25	Free text	Diastolic failure.ti,ab,kw.	96
26	Free text	Systolic failure.ti,ab,kw.	139
27	Free text	systolic dysfunction.ti,ab,kw.	10973
28	Free text	diastolic dysfunction.ti,ab,kw.	13418
29	MeSH	Body Composition/	52390
30	MeSH	Electric Impedance/	21884
31	Free text	Bioimpedance.ti,ab,kw.	6293
32	Free text	Bio-impedance.ti,ab,kw.	724
33	Free text	Bioimpedance analysis.ti,ab,kw.	1571
34	Free text	Bio-impedance analysis.ti,ab,kw.	179
35	Free text	Bioimpedance vector analysis.ti,ab,kw.	92
36	Free text	BIVA.ti,ab,kw.	346

37	Free text	Phase angle.ti,ab,kw.	3918
38	Free text	Electrical impedance.ti,ab,kw.	6223
39	Free text	Lean tissue mass.ti,ab,kw.	705
40	Free text	Lean tissue.ti,ab,kw.	1936
41	Free text	Fat free mass.ti,ab,kw.	10802
42	Free text	Protein energy wasting.ti,ab,kw.	875
43	MeSH	Mortality/	51763
44	MeSH	Morbidity/	35256
45	MeSH	Hospitalization/	150103
46	MeSH	Accidental Falls/	30651
47	MeSH	Frailty/	14116
48	MeSH	Patient Admission/	26875
49	MeSH	Cognition/	143880
50	MeSH	Patient Reported Outcome Measures/	19824
51	MeSH	"Quality of Life"/	312267
52	Free text	Mortality.ti,ab,kw.	1155312
53	Free text	Morbidity.ti,ab,kw.	511398
54	Free text	Dead.ti,ab,kw.	71031
55	Free text	Died.ti,ab,kw.	294488
56	Free text	Survival.ti,ab,kw.	1353269
57	Free text	Hospitalised.ti,ab,kw.	18606
58	Free text	Hospitalized.ti,ab,kw.	158402
59	Free text	Hospitalization.ti,ab,kw.	202508
60	Free text	Hospitalisation.ti,ab,kw.	25527
61	Free text	Admit.ti,ab,kw.	4906
62	Free text	Hospital stay.ti,ab,kw.	115647
63	Free text	Fall.ti,ab,kw.	144949
64	Free text	Frailty.ti,ab,kw.	35648
65	Free text	Symptom burden.ti,ab,kw.	7871
66	Free text	Patient reported outcome measures.ti,ab,kw.	15455
67	Free text	Cognitive function.ti,ab,kw.	65106
68	Free text	Fatigue.ti,ab,kw.	148678
69	Free text	Life participation.ti,ab,kw.	369
70	Free text	Quality of Life.ti,ab,kw.	471297
71	Free text	clinical outcomes.ti,ab,kw.	208534
72	Final combinations (CKD or HF population terms)	1 or 2 or 3 or 4 or 5 or 6 or 7 or 8 or 9 or 10 or 11 or 12 or 13 or 14 or 15 or 16 or 17 or 18 or 19 or 20 or 21 or 22 or 23 or 24 or 25 or 26 or 27 or 28	724899
73	Final combinations (Outcome terms)	43 or 44 or 45 or 46 or 47 or 48 or 49 or 50 or 51 or 52 or 53 or 54 or 55 or 56 or 57 or 58 or 59 or 60 or 61 or 62 or 63 or 64 or 65 or 66 or 67 or 68 or 69 or 70 or 71	4111245
74	Final combinations (BI-MM terms)	29 or 30 or 31 or 32 or 33 or 34 or 35 or 36 or 37 or 38 or 39 or 40 or 41 or 42	85612
75	Final search outcome	72 or 73 or 74	1791

76	**Final search outcome (limited to January 01, 2023)**	**limit 75 to yr="2023 -Current"**	**353**<br>**(See PRISMA Flow Diagram: Figure 1)**

Supplementary Table 3 – Search strategy employed for *EMBASE* during original and updated searches (before duplicate removal).

Number identifying step in search	Type of search term	Search term (as entered exactly into database), along with relevant field tags (if used)	Number of times search identified a result using this search term / combination of terms
ORIGINAL SEARCH:<br>OVID - EMBASE (R) ALL <1974 to June 19, 2023><br>Search conducted 20/06/2023			
1	MeSH	Renal Insufficiency, Chronic/	99930
2	MeSH	Dialysis/ or Renal Dialysis/	170529
3	MeSH	Kidney Transplantation/	133416
4	Free text	Chronic kidney disease.ti,ab,kw.	120780
5	Free text	Chronic renal disease.ti,ab,kw.	5951
6	Free text	Chronic renal impairment.ti,ab,kw.	642
7	Free text	Chronic renal insufficiency.ti,ab,kw.	7196
8	Free text	Kidney insufficiency.ti,ab,kw.	727
9	Free text	End stage kidney disease.ti,ab,kw.	10302
10	Free text	ESKD.ti,ab,kw.	4900
11	Free text	End stage renal disease.ti,ab,kw.	60049
12	Free text	ESRD.ti,ab,kw.	35958
13	Free text	Renal Transplant.ti,ab,kw.	43295
14	Free text	Kidney Transplant.ti,ab,kw.	48497
15	Free text	Dialysis.ti,ab,kw.	184230
16	Free text	Haemodialysis.ti,ab,kw.	24511
17	Free text	Hemodialysis.ti,ab,kw.	110783
18	Free text	Peritoneal dialysis.ti,ab,kw.	39374
19	Free text	Renal replacement therapy.ti,ab,kw.	30128
20	MeSH	Heart Failure/	305068
21	MeSH	Ventricular Dysfunction/	21859
22	Free text	heart failure.ti,ab,kw.	364006
23	Free text	cardiac failure.ti,ab,kw.	19122
24	Free text	congestive cardiac failure.ti,ab,kw.	2328
25	Free text	Diastolic failure.ti,ab,kw.	154
26	Free text	Systolic failure.ti,ab,kw.	225
27	Free text	systolic dysfunction.ti,ab,kw.	19616
28	Free text	diastolic dysfunction.ti,ab,kw.	24357
29	MeSH	Body Composition/	76636
30	MeSH	Electric Impedance/	37812
31	Free text	Bioimpedance.ti,ab,kw.	8480
32	Free text	Bio-impedance.ti,ab,kw.	1165
33	Free text	Bioimpedance analysis.ti,ab,kw.	2114
34	Free text	Bio-impedance analysis.ti,ab,kw.	300

35	Free text	Bioimpedance vector analysis.ti,ab,kw.	177
36	Free text	BIVA.ti,ab,kw.	506
37	Free text	Phase angle.ti,ab,kw.	4225
38	Free text	Electrical impedance.ti,ab,kw.	6720
39	Free text	Lean tissue mass.ti,ab,kw.	937
40	Free text	Lean tissue.ti,ab,kw.	2462
41	Free text	Fat free mass.ti,ab,kw.	12750
42	Free text	Protein energy wasting.ti,ab,kw.	1180
43	MeSH	Mortality/	902330
44	MeSH	Morbidity/	412481
45	MeSH	Hospitalization/	527893
46	MeSH	Accidental Falls/	44500
47	MeSH	Frailty/	27025
48	MeSH	Patient Admission/	262741
49	MeSH	Cognition/	295222
50	MeSH	Patient Reported Outcome Measures/	43866
51	MeSH	"Quality of Life"/	621256
52	Free text	Mortality.ti,ab,kw.	1468131
53	Free text	Morbidity.ti,ab,kw.	683061
54	Free text	Dead.ti,ab,kw.	85237
55	Free text	Died.ti,ab,kw.	432192
56	Free text	Survival.ti,ab,kw.	1768991
57	Free text	Hospitalised.ti,ab,kw.	25335
58	Free text	Hospitalized.ti,ab,kw.	217121
59	Free text	Hospitalization.ti,ab,kw.	290242
60	Free text	Hospitalisation.ti,ab,kw.	36043
61	Free text	Admit.ti,ab,kw.	7381
62	Free text	Hospital stay.ti,ab,kw.	166296
63	Free text	Fall.ti,ab,kw.	167641
64	Free text	Frailty.ti,ab,kw.	39483
65	Free text	Symptom burden.ti,ab,kw.	10855
66	Free text	Patient reported outcome measures.ti,ab,kw.	13426
67	Free text	Cognitive function.ti,ab,kw.	73402
68	Free text	Fatigue.ti,ab,kw.	201494
69	Free text	Life participation.ti,ab,kw.	425
70	Free text	Quality of Life.ti,ab,kw.	603462
71	Free text	clinical outcomes.ti,ab,kw.	255010
72	Final combinations (CKD or HF population terms)	1 or 2 or 3 or 4 or 5 or 6 or 7 or 8 or 9 or 10 or 11 or 12 or 13 or 14 or 15 or 16 or 17 or 18 or 19 or 20 or 21 or 22 or 23 or 24 or 25 or 26 or 27 or 28	1062272
73	Final combinations (Outcome terms)	43 or 44 or 45 or 46 or 47 or 48 or 49 or 50 or 51 or 52 or 53 or 54 or 55 or 56 or 57 or 58 or 59 or 60 or 61 or 62 or 63 or 64 or 65 or 66 or 67 or 68 or 69 or 70 or 71	5799748

74	Final combinations (BI-MM terms)	29 or 30 or 31 or 32 or 33 or 34 or 35 or 36 or 37 or 38 or 39 or 40 or 41 or 42	126154
75	**Final search outcome**	**72 or 73 or 74**	**3873 (See PRISMA Flow Diagram: Figure 1)**
UPDATED SEARCH:<br>OVID - EMBASE (R) ALL <1974 to October 19, 2025><br>Search conducted 20/10/2025			
1	MeSH	Renal Insufficiency, Chronic/	251655
2	MeSH	Dialysis/ or Renal Dialysis/	216157
3	MeSH	Kidney Transplantation/	148861
4	Free text	Chronic kidney disease.ti,ab,kw.	154534
5	Free text	Chronic renal disease.ti,ab,kw.	6568
6	Free text	Chronic renal impairment.ti,ab,kw.	703
7	Free text	Chronic renal insufficiency.ti,ab,kw.	7768
8	Free text	Kidney insufficiency.ti,ab,kw.	847
9	Free text	End stage kidney disease.ti,ab,kw.	14574
10	Free text	ESKD.ti,ab,kw.	7751
11	Free text	End stage renal disease.ti,ab,kw.	68710
12	Free text	ESRD.ti,ab,kw.	41192
13	Free text	Renal Transplant.ti,ab,kw.	46978
14	Free text	Kidney Transplant.ti,ab,kw.	59141
15	Free text	Dialysis.ti,ab,kw.	208345
16	Free text	Haemodialysis.ti,ab,kw.	26882
17	Free text	Hemodialysis.ti,ab,kw.	126966
18	Free text	Peritoneal dialysis.ti,ab,kw.	43521
19	Free text	Renal replacement therapy.ti,ab,kw.	36651
20	MeSH	Heart Failure/	383015
21	MeSH	Ventricular Dysfunction/	25381
22	Free text	heart failure.ti,ab,kw.	433318
23	Free text	cardiac failure.ti,ab,kw.	20597
24	Free text	congestive cardiac failure.ti,ab,kw.	2523
25	Free text	Diastolic failure.ti,ab,kw.	176
26	Free text	Systolic failure.ti,ab,kw.	259
27	Free text	systolic dysfunction.ti,ab,kw.	22457
28	Free text	diastolic dysfunction.ti,ab,kw.	28296
29	MeSH	Body Composition/	98532
30	MeSH	Electric Impedance/	49340
31	Free text	Bioimpedance.ti,ab,kw.	10762
32	Free text	Bio-impedance.ti,ab,kw.	1421
33	Free text	Bioimpedance analysis.ti,ab,kw.	2779
34	Free text	Bio-impedance analysis.ti,ab,kw.	380
35	Free text	Bioimpedance vector analysis.ti,ab,kw.	206
36	Free text	BIVA.ti,ab,kw.	668

37	Free text	Phase angle.ti,ab,kw.	5360
38	Free text	Electrical impedance.ti,ab,kw.	8091
39	Free text	Lean tissue mass.ti,ab,kw.	1065
40	Free text	Lean tissue.ti,ab,kw.	2768
41	Free text	Fat free mass.ti,ab,kw.	15035
42	Free text	Protein energy wasting.ti,ab,kw.	1407
43	MeSH	Mortality/	1022150
44	MeSH	Morbidity/	466144
45	MeSH	Hospitalization/	676292
46	MeSH	Accidental Falls/	56865
47	MeSH	Frailty/	43022
48	MeSH	Patient Admission/	330642
49	MeSH	Cognition/	398391
50	MeSH	Patient Reported Outcome Measures/	85625
51	MeSH	"Quality of Life"/	812111
52	Free text	Mortality.ti,ab,kw.	1747786
53	Free text	Morbidity.ti,ab,kw.	793390
54	Free text	Dead.ti,ab,kw.	95501
55	Free text	Died.ti,ab,kw.	470830
56	Free text	Survival.ti,ab,kw.	2060278
57	Free text	Hospitalised.ti,ab,kw.	30001
58	Free text	Hospitalized.ti,ab,kw.	260808
59	Free text	Hospitalization.ti,ab,kw.	356862
60	Free text	Hospitalisation.ti,ab,kw.	43592
61	Free text	Admit.ti,ab,kw.	8387
62	Free text	Hospital stay.ti,ab,kw.	201324
63	Free text	Fall.ti,ab,kw.	190662
64	Free text	Frailty.ti,ab,kw.	55946
65	Free text	Symptom burden.ti,ab,kw.	15515
66	Free text	Patient reported outcome measures.ti,ab,kw.	21045
67	Free text	Cognitive function.ti,ab,kw.	96422
68	Free text	Fatigue.ti,ab,kw.	249582
69	Free text	Life participation.ti,ab,kw.	596
70	Free text	Quality of Life.ti,ab,kw.	783739
71	Free text	clinical outcomes.ti,ab,kw.	338775
72	Final combinations (CKD or HF population terms)	1 or 2 or 3 or 4 or 5 or 6 or 7 or 8 or 9 or 10 or 11 or 12 or 13 or 14 or 15 or 16 or 17 or 18 or 19 or 20 or 21 or 22 or 23 or 24 or 25 or 26 or 27 or 28	1275463
73	Final combinations (Outcome terms)	43 or 44 or 45 or 46 or 47 or 48 or 49 or 50 or 51 or 52 or 53 or 54 or 55 or 56 or 57 or 58 or 59 or 60 or 61 or 62 or 63 or 64 or 65 or 66 or 67 or 68 or 69 or 70 or 71	6914710
74	Final combinations (BI-MM terms)	29 or 30 or 31 or 32 or 33 or 34 or 35 or 36 or 37 or 38 or 39 or 40 or 41 or 42	160768
75	Final search outcome	72 or 73 or 74	5473

76	**Final search outcome (limited to January 01, 2023)**	**limit 75 to yr="2023 -Current"**	**1293 (See PRISMA Flow Diagram: Figure 1)**

Supplementary Table 4 – Search strategy employed for *AMED* during original and updated searches (before duplicate removal).

**Number identifying step in search**	**Type of search term**	**Search term (as entered exactly into database), along with relevant field tags (if used)**	**Number of times search identified a result using this search term / combination of terms**
ORIGINAL SEARCH:<br>OVID - AMED (Allied and Complementary Medicine) ALL <1985 to May, 2023><br>Search undertaken on 20/06/2023; due to indexing / cataloguing issues with AMED, search results were only captured up until May 2023			
1	MeSH	Renal Insufficiency, Chronic/	0
2	MeSH	Dialysis/ or Renal Dialysis/	239
3	MeSH	Kidney Transplantation/	32
4	MeSH	Kidney disease/	727
5	Free text	Chronic kidney disease.mp.	170
6	Free text	Chronic renal disease.mp.	10
7	Free text	Chronic renal impairment.mp.	0
8	Free text	Chronic renal insufficiency.mp.	12
9	Free text	Kidney insufficiency.mp.	0
10	Free text	End stage kidney disease.mp.	15
11	Free text	ESKD.mp.	6
12	Free text	End stage renal disease.mp.	155
13	Free text	ESRD.mp.	64
14	Free text	Renal Transplant.mp.	18
15	Free text	Kidney Transplant.mp.	25
16	Free text	Dialysis.mp.	434
17	Free text	Haemodialysis.mp.	54
18	Free text	Hemodialysis.mp.	218
19	Free text	Peritoneal dialysis.mp.	37
20	Free text	Renal replacement therapy.mp.	19
21	MeSH	Heart Failure/	516
22	Free text	heart failure.mp.	1027
23	Free text	cardiac failure.mp.	30
24	Free text	congestive cardiac failure.mp.	2
25	Free text	Diastolic failure.mp.	0
26	Free text	Systolic failure.mp.	0
27	Free text	systolic dysfunction.mp.	17
28	Free text	diastolic dysfunction.mp.	15
29	MeSH	Body Composition/	554
30	MeSH	Electric Impedance/	0
31	Free text	Bioimpedance.mp.	37
32	Free text	Bio-impedance.mp.	6
33	Free text	Bioimpedance analysis.mp.	14
34	Free text	Bio-impedance analysis.mp.	2

35	Free text	Bioimpedance vector analysis.mp.	1
36	Free text	BIVA.mp.	2
37	Free text	Phase angle.mp.	30
38	Free text	Electrical impedance.mp.	21
39	Free text	Lean tissue mass.mp.	17
40	Free text	Lean tissue.mp.	32
41	Free text	Fat free mass.mp.	188
42	Free text	Protein energy wasting.mp.	0
43	MeSH	Mortality/	1540
44	MeSH	Morbidity/	239
45	MeSH	Hospitalization/	2155
46	MeSH	Accidental Falls/	2678
47	MeSH	Frailty/	0
48	MeSH	Patient Admission/	236
49	MeSH	Cognition/	2478
50	MeSH	Patient Reported Outcome Measures/	0
51	MeSH	"Quality of Life"/	10941
52	MeSH	Clinical outcomes/	0
53	Free text	Mortality.mp.	3809
54	Free text	Morbidity.mp.	239
55	Free text	Dead.mp.	304
56	Free text	Died.mp.	1491
57	Free text	Survival.mp.	3102
58	Free text	Hospitalised.mp.	133
59	Free text	Hospitalized.mp.	1160
60	Free text	Hospitalization.mp.	183
61	Free text	Hospitalisation.mp.	3056
62	Free text	Clinical outcomes.mp.	1354
63	Free text	Fall.mp.	2311
64	Free text	Frailty.mp.	280
65	Free text	Symptom burden.mp.	142
66	Free text	Patient reported outcome measures.mp.	262
67	Free text	Cognitive function.mp.	781
68	Free text	Fatigue.mp.	7499
69	Free text	Life participation.mp.	48
70	Free text	Quality of Life.mp.	15616
71	Final combinations (CKD or HF population terms)	1 or 2 or 3 or 4 or 5 or 6 or 7 or 8 or 9 or 10 or 11 or 12 or 13 or 14 or 15 or 16 or 17 or 18 or 19 or 20 or 21 or 22 or 23 or 24 or 25 or 26 or 27 or 28	2306
72	Final combinations (Outcome terms)	43 or 44 or 45 or 46 or 47 or 48 or 49 or 50 or 51 or 52 or 53 or 54 or 55 or 56 or 57 or 58 or 59 or 60 or 61 or 62 or 63 or 64 or 65 or 66 or 67 or 68 or 69 or 70	40327
73	Final combinations (BI-MM terms)	29 or 30 or 31 or 32 or 33 or 34 or 35 or 36 or 37 or 38 or 39 or 40 or 41 or 42	763

**74**	**Final search outcome**	**78 and 79 and 80**	**3**<br>**(See PRISMA Flow Diagram: Figure 1)**
UPDATED SEARCH:<br>EBSCO Host - AMED (Allied and Complementary Medicine)<br>Search conducted 21/10/2025			
1	MeSH (free text*)	Renal Insufficiency, Chronic/	15
2	MeSH (free text*)	Dialysis/ or Renal Dialysis/	449
3	MeSH (free text*)	Kidney Transplantation/	56
4	MeSH (free text*)	Kidney disease/	405
5	Free text	Chronic kidney disease	199
6	Free text	Chronic renal disease	46
7	Free text	Chronic renal impairment	1
8	Free text	Chronic renal insufficiency	15
9	Free text	Kidney insufficiency	17
10	Free text	End stage kidney disease	24
11	Free text	ESKD	7
12	Free text	End stage renal disease	175
13	Free text	ESRD	71
14	Free text	Renal Transplant	22
15	Free text	Kidney Transplant	30
16	Free text	Dialysis	449
17	Free text	Haemodialysis	275
18	Free text	Hemodialysis	275
19	Free text	Peritoneal dialysis	41
20	Free text	Renal replacement therapy	21
21	MeSH (free text*)	Heart Failure/	1048
22	Free text	heart failure	1048
23	Free text	cardiac failure	90
24	Free text	congestive cardiac failure	9
25	Free text	Diastolic failure	11
26	Free text	Systolic failure	16
27	Free text	systolic dysfunction	24
28	Free text	diastolic dysfunction	22
29	MeSH (free text*)	Body Composition/	991
30	MeSH (free text*)	Electric Impedance/	12
31	Free text	Bioimpedance	38
32	Free text	Bio-impedance	38
33	Free text	Bioimpedance analysis	15
34	Free text	Bio-impedance analysis	15
35	Free text	Bioimpedance vector analysis	1
36	Free text	BIVA	5
37	Free text	Phase angle	169
38	Free text	Electrical impedance	23

39	Free text	Lean tissue mass	26
40	Free text	Lean tissue	44
41	Free text	Fat free mass	191
42	Free text	Protein energy wasting	26
43	MeSH (free text*)	Mortality/	3866
44	MeSH (free text*)	Morbidity/	2299
45	MeSH (free text*)	Hospitalization/	3383
46	MeSH (free text*)	Accidental Falls/	2790
47	MeSH (free text*)	Frailty/	323
48	MeSH (free text*)	Patient Admission/	952
49	MeSH (free text*)	Cognition/	5792
50	MeSH (free text*)	Patient Reported Outcome Measures/	691
51	MeSH (free text*)	"Quality of Life"/	16173
52	MeSH (free text*)	Clinical outcomes/	3727
53	Free text	Mortality	3866
54	Free text	Morbidity	2299
55	Free text	Dead	300
56	Free text	Died	1489
57	Free text	Survival	3235
58	Free text	Hospitalised	138
59	Free text	Hospitalized	1197
60	Free text	Hospitalization	3383
61	Free text	Hospitalisation	3383
62	Free text	Clinical outcomes	3727
63	Free text	Fall.	4747
64	Free text	Frailty	323
65	Free text	Symptom burden	240
66	Free text	Patient reported outcome measures	691
67	Free text	Cognitive function	1301
68	Free text	Fatigue	7573
69	Free text	Life participation	525
70	Free text	Quality of Life	16729
71	Final combinations (CKD or HF population terms)	1 or 2 or 3 or 4 or 5 or 6 or 7 or 8 or 9 or 10 or 11 or 12 or 13 or 14 or 15 or 16 or 17 or 18 or 19 or 20 or 21 or 22 or 23 or 24 or 25 or 26 or 27 or 28	2082
72	Final combinations (Outcome terms)	43 or 44 or 45 or 46 or 47 or 48 or 49 or 50 or 51 or 52 or 53 or 54 or 55 or 56 or 57 or 58 or 59 or 60 or 61 or 62 or 63 or 64 or 65 or 66 or 67 or 68 or 69 or 70	47839
73	Final combinations (BI-MM terms)	29 or 30 or 31 or 32 or 33 or 34 or 35 or 36 or 37 or 38 or 39 or 40 or 41 or 42	1325
74	Final search outcome	71 and 72 and 73	6
75	**Final search outcome (limited to January 01, 2023)**	**limit 74 to yr="2023 -Current"**	**0**<br>**(See PRISMA Flow Diagram: Figure 1)**

Supplementary Table 5 – Search strategy employed for *APAPsysh / PsychINFO* during original and updated searches (before duplicate removal).

Number identifying step in search	Type of search term	Search term (as entered exactly into database), along with relevant field tags (if used)	Number of times search identified a result using this search term / combination of terms
ORIGINAL SEARCH:<br>EBSCO Host – APA Psych / PyschINFO<br>Search conducted 20/06/2023			
1	Free text	Chronic kidney disease	825
2	Free text	Chronic renal disease	526
3	Free text	Chronic renal impairment	3
4	Free text	Chronic renal insufficiency	29
5	Free text	Kidney insufficiency	71
6	Free text	End stage kidney disease	523
7	Free text	ESKD	12
8	Free text	End stage renal disease	689
9	Free text	ESRD	560
10	Free text	Renal Transplant	426
11	Free text	Kidney Transplant	451
12	Free text	Dialysis	985
13	Free text	Haemodialysis	630
14	Free text	Hemodialysis	630
15	Free text	Peritoneal dialysis	119
16	Free text	Renal replacement therapy	101
17	Free text	heart failure	2361
18	Free text	cardiac failure	1104
19	Free text	congestive cardiac failure	1043
20	Free text	Diastolic failure	92
21	Free text	Systolic failure	209
22	Free text	systolic dysfunction	75
23	Free text	diastolic dysfunction	34
24	MeSH (free text*)	Body Composition/	908
25	MeSH (free text*)	Electric Impedance/	278
26	Free text	Bioimpedance	103
27	Free text	Bio-impedance	103
28	Free text	Bioimpedance analysis	13
29	Free text	Bio-impedance analysis	13
30	Free text	Bioimpedance vector analysis	1
31	Free text	BIVA	4
32	Free text	Phase angle	50
33	Free text	Electrical impedance	278
34	Free text	Lean tissue mass	2

35	Free text	Lean tissue	6
36	Free text	Fat free mass	66
37	Free text	Protein energy wasting.mp.	2
38	MeSH (free text*)	Mortality/	21672
39	MeSH (free text*)	Morbidity/	14909
40	MeSH (free text*)	Hospitalization/	9058
41	MeSH (free text*)	Accidental Falls/	1201
42	MeSH (free text*)	Frailty/	1030
43	MeSH (free text*)	Patient Admission/	137
44	MeSH (free text*)	Patient Reported Outcome Measures/	511
45	MeSH (free text*)	Clinical outcomes/	3591
46	Free text	Mortality	21672
47	Free text	Morbidity	14909
48	Free text	Dead	6171
49	Free text	Died	2774
50	Free text	Survival	14117
51	Free text	Hospitalised	435
52	Free text	Hospitalized	4271
53	Free text	Hospitalization	9058
54	Free text	Hospitalisation	9058
55	Free text	Clinical outcomes	3591
56	Free text	Falls	9775
57	Free text	Frailty	1030
58	Free text	Symptom burden	306
59	Free text	Patient reported outcome measures	511
60	Free text	Cognitive function	19868
61	Free text	Fatigue	6189
62	Free text	Life participation	29
63	Free text	Quality of Life	26140
64	Final combinations (CKD or HF population terms)	1 or 2 or 3 or 4 or 5 or 6 or 7 or 8 or 9 or 10 or 11 or 12 or 13 or 14 or 15 or 16 or 17 or 18 or 19 or 20 or 21 or 22 or 23	4663
65	Final combinations (Outcome terms)	38 or 39 or 40 or 41 or 42 or 43 or 44 or 45 or 46 or 47 or 48 or 49 or 50 or 51 or 52 or 53 or 54 or 55 or 56 or 57 or 58 or 59 or 60 or 61 or 62 or 63	119176
66	Final combinations (BI-MM terms)	24 or 25 or 26 or 27 or 28 or 29 or 30 or 31 or 32 or 33 or 34 or 35 or 36 or 37	1320
**67**	**Final search outcome**	**64 and 65 and 66**	**6**<br>**(See PRISMA Flow Diagram: Figure 1)**
UPDATED SEARCH:<br>EBSCO Host – APA Psych / PyschINFO<br>Search conducted 22/10/2025			
1	MeSH (free text*)	Renal Insufficiency, Chronic/	395
2	MeSH (free text*)	Dialysis/ or Renal Dialysis/	4005

3	MeSH (free text*)	Kidney Transplantation/	858
4	MeSH (free text*)	Kidney disease/	4879
5	Free text	Chronic kidney disease	1596
6	Free text	Chronic renal disease	302
7	Free text	Chronic renal impairment	17
8	Free text	Chronic renal insufficiency	394
9	Free text	Kidney insufficiency	27
10	Free text	End stage kidney disease	239
11	Free text	ESKD	60
12	Free text	End stage renal disease	1270
13	Free text	ESRD	592
14	Free text	Renal Transplant	332
15	Free text	Kidney Transplant	616
16	Free text	Dialysis	4004
17	Free text	Haemodialysis	2240
18	Free text	Hemodialysis	2240
19	Free text	Peritoneal dialysis	325
20	Free text	Renal replacement therapy	194
21	MeSH (free text*)	Heart Failure/	5621
22	Free text	heart failure	5621
23	Free text	cardiac failure	435
24	Free text	congestive cardiac failure	37
25	Free text	Diastolic failure	30
26	Free text	Systolic failure	84
27	Free text	systolic dysfunction	107
28	Free text	diastolic dysfunction	86
29	MeSH (free text*)	Body Composition/	4815
30	MeSH (free text*)	Electric Impedance/	320
31	Free text	Bioimpedance	143
32	Free text	Bio-impedance	143
33	Free text	Bioimpedance analysis	49
34	Free text	Bio-impedance analysis	49
35	Free text	Bioimpedance vector analysis	2
36	Free text	BIVA	12
37	Free text	Phase angle	494
38	Free text	Electrical impedance	128
39	Free text	Lean tissue mass	52
40	Free text	Lean tissue	113
41	Free text	Fat free mass	465
42	Free text	Protein energy wasting	5
43	MeSH (free text*)	Mortality/	58459
44	MeSH (free text*)	Morbidity/	65113
45	MeSH (free text*)	Hospitalization/	55987

46	MeSH (free text*)	Accidental Falls/	4886
47	MeSH (free text*)	Frailty/	4385
48	MeSH (free text*)	Patient Admission/	10815
49	MeSH (free text*)	Cognition/	318333
50	MeSH (free text*)	Patient Reported Outcome Measures/	3067
51	MeSH (free text*)	"Quality of Life"/	123858
52	MeSH (free text*)	Clinical outcomes/	29287
53	Free text	Mortality	58459
54	Free text	Morbidity	65113
55	Free text	Dead	7312
56	Free text	Died	16105
57	Free text	Survival	54635
58	Free text	Hospitalised	1726
59	Free text	Hospitalized	36237
60	Free text	Hospitalization	55987
61	Free text	Hospitalisation	55987
62	Free text	Clinical outcomes	29287
63	Free text	Fall	48699
64	Free text	Frailty	4385
65	Free text	Symptom burden	3636
66	Free text	Patient reported outcome measures	3067
67	Free text	Cognitive function	79992
68	Free text	Fatigue	39305
69	Free text	Life participation	3734
70	Free text	Quality of Life	132861
71	Final combinations (CKD or HF population terms)	1 or 2 or 3 or 4 or 5 or 6 or 7 or 8 or 9 or 10 or 11 or 12 or 13 or 14 or 15 or 16 or 17 or 18 or 19 or 20 or 21 or 22 or 23 or 24 or 25 or 26 or 27 or 28	14242
72	Final combinations (Outcome terms)	43 or 44 or 45 or 46 or 47 or 48 or 49 or 50 or 51 or 52 or 53 or 54 or 55 or 56 or 57 or 58 or 59 or 60 or 61 or 62 or 63 or 64 or 65 or 66 or 67 or 68 or 69 or 70	786318
73	Final combinations (BI-MM terms)	29 or 30 or 31 or 32 or 33 or 34 or 35 or 36 or 37 or 38 or 39 or 40 or 41 or 42	5888
74	Final search outcome	71 and 72 and 73	27
75	**Final search outcome (limited to January 01, 2023)**	**limit 74 to yr="2023 -Current"**	1 **(See PRISMA Flow Diagram: Figure 1)**

In the updated search using EBSCO databases, MeSH terms were incorporated across all database searches, as part of defining the population, prognostic factor measurements and outcomes, to standardise the search strategy. In the original searches, MeSH terms were only used to define prognostic factor measurements and outcomes. Additionally, since the original search, there were changes to the EBSCO interface for advanced searches (which were released in 2024), which could potentially affect citation retrieval. Prior to the EBSCO update, the “Boolean / phrase” search method was employed, whereas after the update, the “Proximity search” method was employed. Given these differences, a sensitivity analysis was performed to ensure the robustness of the original and the updated searches. A sensitivity search, using the updated search terms and the “Proximity search” method, from database inception until the 31st December 2022, identified 23 additional citations. None of these were eligible for inclusion on title / abstract screening. Overall, these findings support the robustness of the original search strategy.

Supplementary Table 6 – Search strategy employed for *CINAHL* during original and updated searches (before duplicate removal).

Number identifying step in search	Type of search term	Search term (as entered exactly into database), along with relevant field tags (if used)	Number of times search identified a result using this search term / combination of terms
ORIGINAL SEARCH:<br>EBSCO Host – CINAHL<br>Search conducted 20/06/2023			
1	Free text	Chronic kidney disease	36133
2	Free text	Chronic renal disease	17998
3	Free text	Chronic renal impairment	67
4	Free text	Chronic renal insufficiency	7783
5	Free text	Kidney insufficiency	4455
6	Free text	End stage kidney disease	25326
7	Free text	ESKD	648
8	Free text	End stage renal disease	29110
9	Free text	ESRD	19317
10	Free text	Renal Transplant	10785
11	Free text	Kidney Transplant	10957
12	Free text	Dialysis	35069
13	Free text	Haemodialysis	25064
14	Free text	Hemodialysis	25064
15	Free text	Peritoneal dialysis	5584
16	Free text	Renal replacement therapy	5992
17	Free text	heart failure	77534
18	Free text	cardiac failure	39231
19	Free text	congestive cardiac failure	38165
20	Free text	Diastolic failure	15
21	Free text	Systolic failure	26
22	Free text	systolic dysfunction	2825
23	Free text	diastolic dysfunction	3127
24	MeSH (free text*)	Body Composition/	25987
25	MeSH (free text*)	Electric Impedance/	2132
26	Free text	Bioimpedance	1485
27	Free text	Bio-impedance	1485
28	Free text	Bioimpedance analysis	364
29	Free text	Bio-impedance analysis	364
30	Free text	Bioimpedance vector analysis	34
31	Free text	BIVA	145
32	Free text	Phase angle	714
33	Free text	Electrical impedance	2132
34	Free text	Lean tissue mass	198

35	Free text	Lean tissue	443
36	Free text	Fat free mass	5186
37	Free text	Protein energy wasting.mp.	313
38	MeSH (free text*)	Mortality/	354690
39	MeSH (free text*)	Morbidity/	124510
40	MeSH (free text*)	Hospitalization/	102914
41	MeSH (free text*)	Accidental Falls/	25927
42	MeSH (free text*)	Frailty/	12922
43	MeSH (free text*)	Patient Admission/	21829
44	MeSH (free text*)	Patient Reported Outcome Measures/	6900
45	MeSH (free text*)	Clinical outcomes/	69501
46	Free text	Mortality	354690
47	Free text	Morbidity	124510
48	Free text	Dead	8741
49	Free text	Died	46262
50	Free text	Survival	232141
51	Free text	Hospitalised	5232
52	Free text	Hospitalized	49342
53	Free text	Hospitalization	102914
54	Free text	Hospitalisation	102914
55	Free text	Clinical outcomes	69501
56	Free text	Falls	59547
57	Free text	Frailty	12922
58	Free text	Symptom burden	2931
59	Free text	Patient reported outcome measures	6900
60	Free text	Cognitive function	48371
61	Free text	Fatigue	55556
62	Free text	Life participation	221
63	Free text	Quality of Life	236225
64	Final combinations (CKD or HF population terms)	1 or 2 or 3 or 4 or 5 or 6 or 7 or 8 or 9 or 10 or 11 or 12 or 13 or 14 or 15 or 16 or 17 or 18 or 19 or 20 or 21 or 22 or 23	160334
65	Final combinations (Outcome terms)	38 or 39 or 40 or 41 or 42 or 43 or 44 or 45 or 46 or 47 or 48 or 49 or 50 or 51 or 52 or 53 or 54 or 55 or 56 or 57 or 58 or 59 or 60 or 61 or 62 or 63	1075550
66	Final combinations (BI-MM terms)	24 or 25 or 26 or 27 or 28 or 29 or 30 or 31 or 32 or 33 or 34 or 35 or 36 or 37	30528
**67**	**Final search outcome**	**64 and 65 and 66**	**666 (See PRISMA Flow Diagram: Figure 1)**
UPDATED SEARCH: EBSCO Host – CINAHL Search conducted 22/10/2025			
1	MeSH (free text*)	Renal Insufficiency, Chronic/	10824
2	MeSH (free text*)	Dialysis/ or Renal Dialysis/	38438

3	MeSH (free text*)	Kidney Transplantation/	15483
4	MeSH (free text*)	Kidney disease/	49815
5	Free text	Chronic kidney disease	40042
6	Free text	Chronic renal disease	10693
7	Free text	Chronic renal impairment	142
8	Free text	Chronic renal insufficiency	10822
9	Free text	Kidney insufficiency	4646
10	Free text	End stage kidney disease	27835
11	Free text	ESKD	737
12	Free text	End stage renal disease	31291
13	Free text	ESRD	20338
14	Free text	Renal Transplant	12343
15	Free text	Kidney Transplant	12750
16	Free text	Dialysis	38438
17	Free text	Haemodialysis	27580
18	Free text	Hemodialysis	27580
19	Free text	Peritoneal dialysis	6206
20	Free text	Renal replacement therapy	6831
21	MeSH (free text*)	Heart Failure/	86373
22	Free text	heart failure	86373
23	Free text	cardiac failure	44709
24	Free text	congestive cardiac failure	40651
25	Free text	Diastolic failure	856
26	Free text	Systolic failure	2042
27	Free text	systolic dysfunction	3609
28	Free text	diastolic dysfunction	3497
29	MeSH (free text*)	Body Composition/	29632
30	MeSH (free text*)	Electric Impedance/	5898
31	Free text	Bioimpedance	1736
32	Free text	Bio-impedance	1736
33	Free text	Bioimpedance analysis	551
34	Free text	Bio-impedance analysis	551
35	Free text	Bioimpedance vector analysis	45
36	Free text	BIVA	185
37	Free text	Phase angle	1305
38	Free text	Electrical impedance	2554
39	Free text	Lean tissue mass	408
40	Free text	Lean tissue	843
41	Free text	Fat free mass	6200
42	Free text	Protein energy wasting	352
43	MeSH (free text*)	Mortality/	382224
44	MeSH (free text*)	Morbidity/	138625
45	MeSH (free text*)	Hospitalization/	116701

46	MeSH (free text*)	Accidental Falls/	28833
47	MeSH (free text*)	Frailty/	17064
48	MeSH (free text*)	Patient Admission/	43449
49	MeSH (free text*)	Cognition/	128493
50	MeSH (free text*)	Patient Reported Outcome Measures/	11573
51	MeSH (free text*)	"Quality of Life"/	263393
52	MeSH (free text*)	Clinical outcomes/	119464
53	Free text	Mortality	382224
54	Free text	Morbidity	138625
55	Free text	Dead	9188
56	Free text	Died	48049
57	Free text	Survival	248296
58	Free text	Hospitalised	5864
59	Free text	Hospitalized	56325
60	Free text	Hospitalization	116701
61	Free text	Hospitalisation	116701
62	Free text	Clinical outcomes	119464
63	Free text	Fall	64609
64	Free text	Frailty	17064
65	Free text	Symptom burden	6069
66	Free text	Patient reported outcome measures	11573
67	Free text	Cognitive function	58648
68	Free text	Fatigue	62684
69	Free text	Life participation	3455
70	Free text	Quality of Life	272244
71	Final combinations (CKD or HF population terms)	1 or 2 or 3 or 4 or 5 or 6 or 7 or 8 or 9 or 10 or 11 or 12 or 13 or 14 or 15 or 16 or 17 or 18 or 19 or 20 or 21 or 22 or 23 or 24 or 25 or 26 or 27 or 28	201036
72	Final combinations (Outcome terms)	43 or 44 or 45 or 46 or 47 or 48 or 49 or 50 or 51 or 52 or 53 or 54 or 55 or 56 or 57 or 58 or 59 or 60 or 61 or 62 or 63 or 64 or 65 or 66 or 67 or 68 or 69 or 70	1299727
73	Final combinations (BI-MM terms)	29 or 30 or 31 or 32 or 33 or 34 or 35 or 36 or 37 or 38 or 39 or 40 or 41 or 42	37126
74	Final search outcome	71 and 72 and 73	790
75	**Final search outcome (limited to January 01, 2023)**	**limit 74 to yr="2023 -Current"**	90 **(See PRISMA Flow Diagram: Figure 1)**

In the updated search using EBSCO databases, MeSH terms were incorporated across all database searches, as part of defining the population, prognostic factor measurements and outcomes, to standardise the search strategy. In the original searches, MeSH terms were only used to define prognostic factor measurements and outcomes. Additionally, since the original search, there were changes to the EBSCO interface for advanced searches (which were released in 2024), which could potentially affect citation retrieval. Prior to the EBSCO update, the “Boolean / phrase” search method was employed, whereas after the update, the “Proximity search” method was employed. Given these differences, a sensitivity analysis was performed to ensure the robustness of the original and the updated searches. A sensitivity search, using the updated search terms and the “Proximity search” method, from database inception until the 31st December 2022, identified 61 additional citations. None of these were eligible for inclusion on title / abstract screening. Overall, these findings support the robustness of the original search strategy.

Supplementary Table 7 – Search strategy employed for *Web of Science Core Collection* during original and updated searches (before duplicate removal).

Number identifying step in search	Type of search term	Search term (as entered exactly into database), along with relevant field tags (if used)	Number of times search identified a result using this search term / combination of terms
ORIGINAL SEARCH:<br>Web of Science Core Collection<br>Search conducted 20/06/2023			
1	MeSH (free text*)	TS=(Renal Insufficiency, Chronic/)	12151
2	MeSH (free text*)	TS=(Dialysis/ or Renal Dialysis/)	137818
3	MeSH (free text*)	TS=(Kidney Transplantation/)	84194
4	Free text	TS=(Chronic kidney disease)	118262
5	Free text	TS=(Chronic renal disease)	94266
6	Free text	TS=(Chronic renal impairment)	7173
7	Free text	TS=(Chronic renal insufficiency)	12151
8	Free text	TS=(Kidney insufficiency)	11934
9	Free text	TS=(End stage kidney disease)	31760
10	Free text	TS=(ESKD)	2516
11	Free text	TS=(End stage renal disease)	50137
12	Free text	TS=(ESRD)	19618
13	Free text	TS=(Renal Transplant)	76195
14	Free text	TS=(Kidney Transplant)	74225
15	Free text	TS=(Dialysis)	137818
16	Free text	TS=(Haemodialysis)	14554
17	Free text	TS=(Hemodialysis)	105396
18	Free text	TS=(Peritoneal dialysis)	33101
19	Free text	TS=(Renal replacement therapy)	24251
20	MeSH (free text*)	TS=(Heart Failure/)	340882
21	MeSH (free text*)	TS=(Ventricular Dysfunction/)	81001
22	Free text	TS=(heart failure)	348882
23	Free text	TS=(cardiac failure)	156733
24	Free text	TS=(congestive cardiac failure)	19959
25	Free text	TS=(Diastolic failure)	32001
26	Free text	TS=(Systolic failure)	42677
27	Free text	TS=(systolic dysfunction)	37726
28	Free text	TS=(diastolic dysfunction)	32909
29	MeSH (free text*)	TS=(Body Composition/)	151245
30	MeSH (free text*)	TS=(Electric Impedance/)	52280
31	Free text	TS=(Bioimpedance)	8289
32	Free text	TS=(Bio-impedance)	1142

33	Free text	TS=(Bioimpedance analysis)	4355
34	Free text	TS=(Bio-impedance analysis)	476
35	Free text	TS=(Bioimpedance vector analysis)	418
36	Free text	TS=(BIVA)	329
37	Free text	TS=(Phase angle)	143704
38	Free text	TS=(Electrical impedance)	52280
39	Free text	TS=(Lean tissue mass)	6462
40	Free text	TS=(Lean tissue)	12904
41	Free text	TS=(Fat free mass)	17123
42	Free text	TS=(Protein energy wasting)	4902
43	MeSH (free text*)	TS=(Mortality/)	1288838
44	MeSH (free text*)	TS=(Morbidity/)	465138
45	MeSH (free text*)	TS=(Hospitalization/)	211758
46	MeSH (free text*)	TS=(Accidental Falls/)	4204
47	MeSH (free text*)	TS=(Frailty/)	35601
48	MeSH (free text*)	TS=(Patient Admission/)	202282
49	MeSH (free text*)	TS=(Cognition/)	211828
50	MeSH (free text*)	TS=(Patient Reported Outcome Measures/)	104064
51	MeSH (free text*)	TS=("Quality of Life"/)	709495
52	Free text	TS=(Mortality)	1288838
53	Free text	TS=(Morbidity)	465138
54	Free text	TS=(Dead)	141500
55	Free text	TS=(Died)	407187
56	Free text	TS=(Survival)	1526059
57	Free text	TS=(Hospitalised)	144632
58	Free text	TS=(Hospitalized)	144632
59	Free text	TS=(Hospitalization)	211758
60	Free text	TS=(Hospitalisation)	211758
61	Free text	TS=(Admit)	279094
62	Free text	TS=(Hospital stay)	156033
63	Free text	TS=(Fall)	477248
64	Free text	TS=(Frailty)	35601
65	Free text	TS=(Symptom burden)	44237
66	Free text	TS=(Patient reported outcome measures)	104064
67	Free text	TS=(Cognitive function)	217770
68	Free text	TS=(Fatigue)	313126
69	Free text	TS=(Life participation)	53051
70	Free text	TS=(Quality of Life)	709495
71	Free text	TS=(clinical outcomes)	954861
72	Final combinations (CKD or HF population terms)	1 or 2 or 3 or 4 or 5 or 6 or 7 or 8 or 9 or 10 or 11 or 12 or 13 or 14 or 15 or 16 or 17 or 18 or 19 or 20 or 21 or 22 or 23 or 24 or 25 or 26 or 27 or 28	455052

73	Final combinations (Outcome terms)	43 or 44 or 45 or 46 or 47 or 48 or 49 or 50 or 51 or 52 or 53 or 54 or 55 or 56 or 57 or 58 or 59 or 60 or 61 or 62 or 63 or 64 or 65 or 66 or 67 or 68 or 69 or 70 or 71	5841804
74	Final combinations (BI-MM terms)	29 or 30 or 31 or 32 or 33 or 34 or 35 or 36 or 37 or 38 or 39 or 40 or 41 or 42	369649
75	**Final search outcome**	**72 or 73 or 74**	**3628**<br>**(See PRISMA Flow Diagram: Figure 1)**
UPDATED SEARCH:<br>Web of Science Core Collection<br>Search conducted 23/10/2025			
1	MeSH (free text*)	TS=(Kidney disease/)	309343
2	MeSH (free text*)	TS=(Renal Insufficiency, Chronic/)	13781
3	MeSH (free text*)	TS=(Dialysis/ or Renal Dialysis/)	163849
4	MeSH (free text*)	TS=(Kidney Transplantation/)	97171
5	Free text	TS=(Chronic kidney disease)	148911
6	Free text	TS=(Chronic renal disease)	110894
7	Free text	TS=(Chronic renal impairment)	8539
8	Free text	TS=(Chronic renal insufficiency)	13781
9	Free text	TS=(Kidney insufficiency)	13750
10	Free text	TS=(End stage kidney disease)	38490
11	Free text	TS=(ESKD)	4331
12	Free text	TS=(End stage renal disease)	57362
13	Free text	TS=(ESRD)	22047
14	Free text	TS=(Renal Transplant)	84514
15	Free text	TS=(Kidney Transplant)	88393
16	Free text	TS=(Dialysis)	163849
17	Free text	TS=(Haemodialysis)	16457
18	Free text	TS=(Hemodialysis)	120191
19	Free text	TS=(Peritoneal dialysis)	37276
20	Free text	TS=(Renal replacement therapy)	29193
21	MeSH (free text*)	TS=(Heart Failure/)	405216
23	Free text	TS=(heart failure)	405216
24	Free text	TS=(cardiac failure)	185633
25	Free text	TS=(congestive cardiac failure)	22505
26	Free text	TS=(Diastolic failure)	37144
27	Free text	TS=(Systolic failure)	48717
28	Free text	TS=(systolic dysfunction)	43105
29	Free text	TS=(diastolic dysfunction)	37722
30	MeSH (free text*)	TS=(Body Composition/)	183480
31	MeSH (free text*)	TS=(Electric Impedance/)	23171
32	Free text	TS=(Bioimpedance)	10086
33	Free text	TS=(Bio-impedance)	1314
34	Free text	TS=(Bioimpedance analysis)	5411
35	Free text	TS=(Bio-impedance analysis)	540

36	Free text	TS=(Bioimpedance vector analysis)	512
37	Free text	TS=(BIVA)	428
38	Free text	TS=(Phase angle)	170818
39	Free text	TS=(Electrical impedance)	62355
40	Free text	TS=(Lean tissue mass)	7450
41	Free text	TS=(Lean tissue)	15030
42	Free text	TS=(Fat free mass)	20174
43	Free text	TS=(Protein energy wasting)	6419
44	MeSH (free text*)	TS=(Mortality/)	1579289
45	MeSH (free text*)	TS=(Morbidity/)	550847
46	MeSH (free text*)	TS=(Hospitalization/)	269009
47	MeSH (free text*)	TS=(Accidental Falls/)	5013
48	MeSH (free text*)	TS=(Frailty/)	50977
49	MeSH (free text*)	TS=(Clinical outcomes/)	1241732
50	MeSH (free text*)	TS=(Patient Admission/)	250338
51	MeSH (free text*)	TS=(Cognition/)	256777
52	MeSH (free text*)	TS=(Patient Reported Outcome Measures/)	133179
53	MeSH (free text*)	TS=("Quality of Life"/)	665214
54	Free text	TS=(Mortality)	1579289
55	Free text	TS=(Morbidity)	550847
56	Free text	TS=(Dead)	161181
57	Free text	TS=(Died)	483895
58	Free text	TS=(Survival)	1831363
59	Free text	TS=(Hospitalised)	181331
60	Free text	TS=(Hospitalized)	181331
61	Free text	TS=(Hospitalization)	269009
62	Free text	TS=(Hospitalisation)	269009
65	Free text	TS=(Fall)	603990
66	Free text	TS=(Frailty)	50977
67	Free text	TS=(Symptom burden)	60294
68	Free text	TS=(Patient reported outcome measures)	133179
69	Free text	TS=(Cognitive function)	269394
70	Free text	TS=(Fatigue)	379193
71	Free text	TS=(Life participation)	66413
72	Free text	TS=(Quality of Life)	888921
73	Free text	TS=(clinical outcomes)	1241732
73	Final combinations (CKD or HF population terms)	1 or 2 or 3 or 4 or 5 or 6 or 7 or 8 or 9 or 10 or 11 or 12 or 13 or 14 or 15 or 16 or 17 or 18 or 19 or 20 or 21 or 22 or 23 or 24 or 25 or 26 or 27 or 28 or 29	1082350
74	Final combinations (Outcome terms)	44 or 45 or 46 or 47 or 48 or 49 or 50 or 51 or 52 or 53 or 54 or 55 or 56 or 57 or 58 or 59 or 60 or 61 or 62 or 63 or 64 or 65 or 66 or 67 or 68 or 69 or 70 or 71 or 42	6945926
75	Final combinations (BI-MM terms)	30 or 31 or 32 or 33 or 34 or 35 or 36 or 37 or 38 or 39 or 40 or 41 or 42 or 43	459107

76	Final search outcome	73 and 74 and 75	4570
77	**Final search outcome (limited to January 01, 2023)**	**limit 76 to yr="2023 -Current"**	**977 (See PRISMA Flow Diagram: Figure 1)**
Minor variations existed between the search strategies employed for the Web of Science Core Collection. The original search included *TS=(ventricular dysfunction/)* and did not include *TS=(kidney disease/)*, as part of the population search terms used. Additionally, the terms *TS=(Admit)* and *TS=(Hospital stay)* were not included in the updated search. Comparing citations retrieved in the updated search (n=4570) with a sensitivity search that used the exact search terms used in the original search (n=4451), 62 citations unique to the sensitivity search were identified. None of these satisfied the inclusion criteria for this study on title / abstract screening. These findings demonstrate the robustness of the updated search strategy.			

Supplementary Table 8 – Search strategy employed for *CENTRAL* during original and updated searches (before duplicate removal).

Number identifying step in search	Type of search term	Search term (as entered exactly into database), along with relevant field tags (if used)	Number of times search identified a result using this search term / combination of terms
ORIGINAL SEARCH:<br>Cochrane Central Register of Controlled Trials (CENTRAL)<br>Search conducted 20/06/2023			
1	Free text	Chronic kidney disease:ti,ab,kw.	15235
2	Free text	Chronic renal disease:ti,ab,kw.	13117
3	Free text	Chronic renal impairment:ti,ab,kw.	1123
4	Free text	Chronic renal insufficiency:ti,ab,kw.	4908
5	Free text	Kidney insufficiency:ti,ab,kw.	5754
6	Free text	End stage kidney disease:ti,ab,kw.	5553
7	Free text	ESKD:ti,ab,kw.	357
8	Free text	End stage renal disease:ti,ab,kw.	7102
9	Free text	ESRD:ti,ab,kw.	2308
10	Free text	Renal Transplant:ti,ab,kw.	8341
11	Free text	Kidney Transplant:ti,ab,kw.	8059
12	Free text	Dialysis:ti,ab,kw.	16758
13	Free text	Haemodialysis:ti,ab,kw.	14113
14	Free text	Hemodialysis:ti,ab,kw.	14113
15	Free text	Peritoneal dialysis:ti,ab,kw.	2652
16	Free text	Renal replacement therapy:ti,ab,kw.	3955
17	Free text	heart failure:ti,ab,kw.	44418
18	Free text	cardiac failure:ti,ab,kw.	19613
19	Free text	congestive cardiac failure:ti,ab,kw.	2702
20	Free text	Diastolic failure:ti,ab,kw.	5324
21	Free text	Systolic failure:ti,ab,kw.	8698
22	Free text	Systolic dysfunction:ti,ab,kw.	4373
23	Free text	Diastolic dysfunction:ti,ab,kw.	3225
24	Free text	Ventricular dysfunction:ti,ab,kw.	6556
25	Free text	Bioimpedance:ti,ab,kw.	1346
26	Free text	Bio-impedance:ti,ab,kw.	222
27	Free text	Bioimpedance vector analysis:ti,ab,kw.	27
28	Free text	BIVA:ti,ab,kw.	35
29	Free text	Phase angle:ti,ab,kw.	1439
30	Free text	Electrical impedance:ti,ab,kw.	738
31	Free text	Fat free mass:ti,ab,kw.	3815
32	Free text	Lean tissue mass:ti,ab,kw.	1376
33	Free text	Lean tissue:ti,ab,kw.	1735
34	Free text	Mortality:ti,ab,kw.	112347

35	Free text	Morbidity:ti,ab,kw.	44897
36	Free text	Hospitalization:ti,ab,kw.	51496
37	Free text	Accidental falls:ti,ab,kw.	2215
38	Free text	Frailty:ti,ab,kw.	3109
39	Free text	Patient admission:ti,ab,kw.	16662
40	Free text	Cognition:ti,ab,kw.	32821
41	Free text	Patient reported outcome measures:ti,ab,kw.	14711
42	Free text	Quality of Life:ti,ab,kw.	154480
43	Free text	Dead:ti,ab,kw.	2173
44	Free text	Died:ti,ab,kw.	15174
45	Free text	Survival:ti,ab,kw.	127492
46	Free text	Hospitalised:ti,ab,kw.	20212
47	Free text	Hospitalized:ti,ab,kw.	20212
48	Free text	Hospitalisation:ti,ab,kw.	51558
49	Free text	Admit:ti,ab,kw.	395
50	Free text	Hospital stay:ti,ab,kw.	39647
51	Free text	Fall:ti,ab,kw.	14987
52	Free text	Symptom burden:ti,ab,kw.	3855
53	Free text	Cognitive function:ti,ab,kw.	25236
54	Free text	Fatigue:ti,ab,kw.	42800
55	Free text	Life participation:ti,ab,kw.	10079
56	Free text	clinical outcomes:ti,ab,kw.	216065
57	Final combinations (CKD or HF population terms)	1 or 2 or 3 or 4 or 5 or 6 or 7 or 8 or 9 or 10 or 11 or 12 or 13 or 14 or 15 or 16 or 17 or 18 or 19 or 20 or 21 or 22 or 23 or 24	92860
58	Final combinations (Outcome terms)	25 or 26 or 27 or 28 or 29 or 30 or 31 or 32	603367
59	Final combinations (BI-MM terms)	33 or 34 or 35 or 36 or 37 or 38 or 39 or 40 or 41 or 42 or 43 or 44 or 45 or 46 or 47 or 48 or 49 or 50 or 51 or 52 or 53 or 54 or 55 or 56	8574
60	**Final search outcome**	**57 and 58 and 59**	**338 (See PRISMA Flow Diagram: Figure 1)**
UPDATED SEARCH: Cochrane Central Register of Controlled Trials (CENTRAL) Search conducted 23/10/2023			
1	Free text	Chronic kidney disease:ti,ab,kw.	18746
2	Free text	Chronic renal disease:ti,ab,kw.	15101
3	Free text	Chronic renal impairment:ti,ab,kw.	1276
4	Free text	Chronic renal insufficiency:ti,ab,kw.	5873
5	Free text	Kidney insufficiency:ti,ab,kw.	6845
6	Free text	End stage kidney disease:ti,ab,kw.	6407
7	Free text	ESKD:ti,ab,kw.	536
8	Free text	End stage renal disease:ti,ab,kw.	7992
9	Free text	ESRD:ti,ab,kw.	2558

10	Free text	Renal Transplant:ti,ab,kw.	8835
11	Free text	Kidney Transplant:ti,ab,kw.	8835
12	Free text	Dialysis:ti,ab,kw.	19047
13	Free text	Haemodialysis:ti,ab,kw.	15946
14	Free text	Hemodialysis:ti,ab,kw.	15946
15	Free text	Peritoneal dialysis:ti,ab,kw.	2902
16	Free text	Renal replacement therapy:ti,ab,kw.	4681
17	Free text	heart failure:ti,ab,kw.	50179
18	Free text	cardiac failure:ti,ab,kw.	22289
19	Free text	congestive cardiac failure:ti,ab,kw.	2729
20	Free text	Diastolic failure:ti,ab,kw.	6018
21	Free text	Systolic failure:ti,ab,kw.	9766
23	Free text	Systolic dysfunction:ti,ab,kw.	4826
24	Free text	Diastolic dysfunction:ti,ab,kw.	3520
25	Free text	Ventricular dysfunction:ti,ab,kw.	7165
26	Free text	Bioimpedance:ti,ab,kw.	1652
27	Free text	Bio-impedance:ti,ab,kw.	261
28	Free text	Bioimpedance vector analysis:ti,ab,kw.	32
29	Free text	BIVA:ti,ab,kw.	42
30	Free text	Phase angle:ti,ab,kw.	1758
31	Free text	Electrical impedance:ti,ab,kw.	959
32	Free text	Fat free mass:ti,ab,kw.	4441
33	Free text	Lean tissue mass:ti,ab,kw.	1526
34	Free text	Lean tissue:ti,ab,kw.	1926
35	Free text	Mortality:ti,ab,kw.	128893
36	Free text	Morbidity:ti,ab,kw.	49766
37	Free text	Hospitalization:ti,ab,kw.	61305
38	Free text	Accidental falls:ti,ab,kw.	2665
39	Free text	Frailty:ti,ab,kw.	4553
40	Free text	Patient admission:ti,ab,kw.	19326
41	Free text	Cognition:ti,ab,kw.	40366
42	Free text	Patient reported outcome measures:ti,ab,kw.	17842
43	Free text	Quality of Life:ti,ab,kw.	192065
44	Free text	Dead:ti,ab,kw.	16303
45	Free text	Died:ti,ab,kw.	2489
46	Free text	Survival:ti,ab,kw.	145173
47	Free text	Hospitalised:ti,ab,kw.	61305
48	Free text	Hospitalized:ti,ab,kw.	23600
49	Free text	Hospitalisation:ti,ab,kw.	23600
50	Free text	Admit:ti,ab,kw.	431
51	Free text	Hospital stay:ti,ab,kw.	47568
52	Free text	Fall:ti,ab,kw.	16932
53	Free text	Symptom burden:ti,ab,kw.	5100

54	Free text	Cognitive function:ti,ab,kw.	32065
55	Free text	Fatigue:ti,ab,kw.	51311
56	Free text	Life participation:ti,ab,kw.	12778
57	Free text	clinical outcomes:ti,ab,kw.	270270
58	Final combinations (CKD or HF population terms)	1 or 2 or 3 or 4 or 5 or 6 or 7 or 8 or 9 or 10 or 11 or 12 or 13 or 14 or 15 or 16 or 17 or 18 or 19 or 20 or 21 or 22 or 23 or 24	104870
59	Final combinations (Outcome terms)	25 or 26 or 27 or 28 or 29 or 30 or 31 or 32	10160
60	Final combinations (BI-MM terms)	33 or 34 or 35 or 36 or 37 or 38 or 39 or 40 or 41 or 42 or 43 or 44 or 45 or 46 or 47 or 48 or 49 or 50 or 51 or 52 or 53 or 54 or 55 or 56	722360
61	Final search outcome	58 and 59 and 60	410
62	**Final search outcome (limited to January 01, 2023)**	61 with Cochrane Library publication date Between Jan 2023 and Oct 2025	**109 (See PRISMA Flow Diagram: Figure 1)**

Supplementary Table 9 – Operationalisation criteria for the QUIPS tool (risk of bias assessments).

Risk of Bias (ROB) Domains	Signalling items / questions for consideration when assessing ROB.	Yes – this question is clearly answered.	Partly – this question is partially answered.	No – this question is not answered.	Unclear – it is unclear from the study whether this question has been answered.
**1. Study Participation (SP)**	**Optimal study - the study sample adequately represents the population of interest.**				
	A. Is there a clear description of the population of interest / source population for the study?	Study clearly identifies population of interest for research as CKD (G3A-5, D-KD or KTR) or chronic heart failure, and provides clear definitions for classification of patients within this population (i.e. severity or stage of CKD is clearly defined, with defined eGFR cut points in the case of a non-dialysis CKD population).<br><br>Where these details are not clear in the specified citation, references are made to the earlier study population where these details are clearly provided.	The study identifies source population in general terms (i.e. "CKD" or "heart failure", but makes no reference to the definitions used to classify either CKD or chronic heart failure (i.e. CKD is not defined using an eGFR cut point of < 60ml/min/1.73m^2 or CKD3A+, or in case of heart failure, no diagnostic criteria used to define heart failure using echocardiogram data / BNP data etc).	Study doesn't clearly identify the population of interest for the research (this may well lead to exclusion of study, as it is essential that the population of interest is specified to some degree for inclusion within the systematic review).	If study is unclear – the same concerns about exclusion should be addressed given the population of interest needs to be clear.
	B. Is there clearly defined inclusion and exclusion criteria on which eligibility to participate in the study is determined?	Study clearly describes inclusion and exclusion criteria, and clearly specifies diagnostic cut points for these criteria if relevant.	Studies discuss either inclusion or exclusion criteria, or doesn't clearly define diagnostic cut points if relevant.	Study doesn't report any inclusion / exclusion criteria.	N/A.
	C. Is the time period of recruitment clearly defined?	The time period of recruitment is clearly defined, including start / end dates of recruitment. Alternatively, a start date and a duration of recruitment (with definition of this duration in days / months / years) would specify a similar clear recruitment period.	Some aspects of the time period of recruitment are reported (i.e. duration, but not start date).	No details about time period of recruitment is given.	N/A.
	D. Is the place of recruitment clearly defined?	The place of recruitment is clearly described. This will include reference to the countries the research was conducted in, the number of centres included and the cities / locations of these centres.	Partial details given – i.e. country but no mention of city, or doesn't report number of centres.	No details about the place of recruitment are provided.	N/A

	E. Is there an adequate description of the sampling frame (process by which study sample was chosen from population of interest) and process of recruitment?	How patients were identified, approached to participate and were consented is clearly specified in the paper. There were no systematic differences in the way patients were approached to participate in the study.	Partial details are provided.	No details provided about the process of recruitment / sampling frame.	N/A
	F. Is there adequate participation in the study by eligible persons?	Of the eligible population, did ≥ 70% participate in the final study and were counted in the final study sample.	< 70% study participation is reported, but this is justified in the study. Additionally, there may be measures taken to minimise the effect of selection bias on the measures of effect.	< 70% study participation is reported with no attempt to justify this.	Insufficient information presented to make a judgement on this question.
	G. Is there an adequate description of the baseline characteristics of the study participants (i.e. the study sample)?	Are the study sample baseline characteristics clearly described?<br><br>Given the hypothesis for our research question, we need to ensure that at least 5 of these core baseline characteristics are reported for CKD studies (* variables needed for the study to be counted as low ROB, with either comorbidity score or diabetic status being reported):<br>- Age.*<br>- Sex.*<br>- Ethnicity.<br>- Comorbidity score.*<br>- Cardiovascular disease.<br>- Diabetes mellitus.*<br>- Smoking status.<br>- CKD stage (for CKD studies), eGFR (for CKD or transplant studies) or RRT Modality (for dialysis studies).<br>- Residual renal function.<br>- Inflammatory status (CRP / IL6).<br>- Dialysis efficiency indices (URR / KtV).<br>- Nutritional indices (SGA / MIS).<br>- Serum albumin.	Study reports 5 or more of the core baseline characteristics but fails to include all 3 essential baseline characteristics (age, sex and diabetes mellitus / comorbidity score).	Study reports less than 5 core baseline characteristics.	Baseline characteristics are not reported in the main text or in tabular form.

		- Overhydration (if reported).<br><br>For heart failure studies):<br>- Age.*<br>- Sex.*<br>- Ethnicity.<br>- Comorbidity score.**<br>- Diabetic status.**<br>- Blood pressure recordings.<br>- Smoking status.<br>- Echocardiographic data (LVEF / RVEF / LVM etc).<br>- Renal function.<br>- Inflammatory status (CRP / IL6).<br>- Nutritional indices used in heart failure<br>- Serum albumin.<br>- Overhydration (if reported).			
**2. Study Attrition (SA)**	**Optimal study - the study participants completing the study adequately represents the original study sample.**				
	A. Are there sufficient study participants completing the study?	Of the study participants, ≥ 70% completed the study.	N/A	< 70% of study participants completed the study.	No attrition data is reported, and therefore it is not possible to say whether sufficient study participants completed the study and whether any important differences were present between those completing and not completing the study.
	B. Are there attempts to collect data on study participants who did not complete the study?	There is a clear description of the methods used to contact those who were lost to follow up, sufficient to allow for another investigator to copy the method.	There are some details provided, but they could not be easily repeated by another investigator.	There is no description of what was done in the study to contact those lost to follow up.	
	C. Is there an adequate description of the characteristics of study participants who did not complete the study?	There is a clear description of the characteristics of participants that did not complete the study. This may include the core baseline characteristics and descriptive statistics to compare these two groups of study participants	There are brief descriptions of the differences between those completing and not completing the study.	There is no description of the characteristics of participants that failed to complete the study.	
	D. Are reasons provided for participants not completing the study?	There is a clear summary of reasons for not completing the study, either in the text of the paper or in tabular form.	There is some detail given about reasons for not completing the study, but these are incomplete.	There is no reporting of reasons for not completing the study.	
	E. Can we say there are no important differences between study participants who completed the study and those that did not?	We can, given the above data, say with confidence that there are no differences between those participants completing and not completing the study.	We can be reasonably confidence that there are no differences between participants completing and not completing the study.	There are differences between study participants completing and not completing the study.	

3. Prognostic Factor Measurement (PFM)	Optimal study - the prognostic factor is measured in the same way for all study participants.				
	A. Is a clear definition or description of the PFM provided?	A clear definition for the PFM (BI measure) is provided, including the specific measure being used along with appropriate units where appropriate.	The PFM is defined, but the units are not specified or cut points, if used, are not clearly defined.	There is no clear definition of the PFM used in the study (this may well lead to exclusion of study, as it is essential that the PFM is specified to some degree for inclusion within the systematic review).	If study is unclear – the same concerns about exclusion should be addressed given the population of interest needs to be clear.
	B. Is the method for PFM measurement adequately valid / reliable?	A validated measure of the PFM (BI measure) was conducted (i.e. PA, BIVA, LTMI, BCM measures etc), including the specification of a recognised bioimpedance device (the bioimpedance device used is specified according to the model, not just the brand name).	A validated measure of PFM (BI measure) was conducted, and some detail is given about the device used to conduct the measurement.	A non-validated measure of the PFM was taken. It is reasonable to assume this if no specifics about the bioimpedance device used are given.	It is unclear what method was used to measure the PFM; although, in such circumstances, it could be argued that the study is at high risk of biased estimates of effect with respect to the PFM.
	C. Are continuous values for the PFM reported appropriately, or do they make use of appropriate cut points?	Continuous values for the BI measure are clearly reported at baseline and within analyses, including appropriate measures of location and dispersion (mean / median, standard deviation / interquartile range). In the case of BI-LTM cut offs being used (for instance, if used to stratify groups for subsequent observation), there is a clear justification for this from the data or from previously published observations. Treatment and reporting of BI data is in accordance with theoretical considerations when reporting BI data and is not solely on the basis of statistical considerations (an example would be correct interpretation of the directionality of PA data).	There is some reporting of continuous variables, but not to the extent we see with a clear "yes" to this signalling question / item.	There are no BI measurement values reported in the study, no measures of dispersion or incorrect treatment of BI data based on theoretical considerations (for example the incorrect directionality of PA data on the measure of effect).	N/A.
	D. Is the PFM measurement method the same for all participants?	PFM (BI) measurements are taken in the same way for all study participants.	N/A.	PFM (BI) measurements are taken in a non-systematic way across the study.	It is unclear what method was used to measure the PFM (BI) in the study.
	E. Is there an adequate proportion of study	≥ 90% of study participants have at least one value reported for the PFM (BI).	< 90% of the study population have reported BI data, but justifications	< 90% of the study population have reported	It is unclear what proportion of the study population have BI data reported in the study.

	participants with complete PFM data reported?		are given (including justifiable and valid attempts to address this: see signalling item below).	BI data, and no justification is given.	
	F. In the event of missing data, are imputation methods described and implemented?	When imputation methods are used to handle missing data, the research question / methodology / nature of the data drives the method used (single vs multiple, random vs non-random methods). Furthermore, if completed case analysis is used, this is justified.	Imputation methods are used but only some details of the methods are stated. However, the method ultimately chosen appears appropriate.	Imputation methods are used to handle missing data but are based on untenable assumptions about the data missing mechanism. Furthermore, no specific justifications for the method applied are used.	It is unclear how missing data is handled.
**4. Outcome Measurement (OM)**	**Optimal study - The outcome of interest is measured in a similar way for all participants.**				
	A. Is a clear definition of the outcome variable (or variables) provided in the study?	The outcome measure is clearly defined (mortality, all-cause mortality, frailty surrogates), including appropriate definitions / subclassification of outcomes where appropriate.	The outcome measure is defined but there could be more granularity in the definition to ensure clarity.	The outcome measure is not clearly defined (this may well lead to exclusion of study, as it is essential that the OM is specified to some degree for inclusion within the systematic review).	If study is unclear – the same concerns about exclusion should be addressed given the outcome measure needs to be clear.
	B. Is the method of determining the outcome adequately valid and reliable?	The methods for determining the outcome measure are valid and have precedent throughout the literature (retrospective review of notes, follow up interviews etc).	There is some detail / validity in the method for determining outcome, but more detail is needed to ensure there is a robust definition.	The methods for determining the outcome are not valid or not justified. There is risk of recall bias in the methods used (if self-reported outcomes such as hospitalisation). In the case of cardiovascular mortality, there are no validated methods for confirming this (i.e. postmortem).	It is unclear what methods were used to determine the outcome measure, and whether as it is not specified in the paper.
	C. Is the method and setting of outcome measurement the same for all study participants?	The same method for outcome measurement is used for all study participants.	N/A	The same method for outcome measurement is not the same for all study participants.	It is unclear whether the method for determining the outcome measure is the same across all participants in the study.
**5. Study Confounding (SC)**	**Optimal study – important potential confounding variables are appropriately accounted for in the study design and analysis**				

A. Are all potential covariates that may affect the study outcome measured / reported?	Are all the relevant covariates that may affect the measure of effect between the PFM (BI) and the outcomes of interest reported in the study?<br><br>Given the hypothesis for our research question, we need to ensure that data from at least 5 of these important covariates is reported within the study and accounted for in the survival analyses (with *being essential covariates for the study to be counted as low ROB, with ether diabetes mellitus or comorbidity score being adjusted for):<br>- Age.*<br>- Sex.*<br>- Ethnicity.<br>- Comorbidity score.*<br>- Diabetes mellitus.*<br>- Other stated comorbidities (i.e. CVD)<br>- Smoking status.<br>- CKD stage (for CKD studies), eGFR (for CKD or transplant studies) or RRT Modality (for dialysis studies).<br>- Residual renal function.<br>- Vintage<br>- Inflammatory status (CRP / IL6).<br>- Dialysis efficiency indices (URR / KtV).<br>- Nutritional indices (SGA / MIS).<br>- Serum albumin.<br>- Overhydration (if reported).<br><br>For heart failure studies:<br>- Age.*<br>- Sex.*<br>- Ethnicity.<br>- Comorbidity score.*<br>- Diabetic status.*<br>- Smoking status.<br>- BP recordings	Study reports 5 or more core covariates but fails to include all 3 essential covariates (age, sex, comorbidity score or diabetes mellitus).	Study reports less than 5 core covariates.	Covariate data is not reported in the main text or in tabular form, and therefore it is not possible to reach a conclusion.

	- Echocardiographic data (LVEF / RVEF / LVM etc).<br>- Renal function.<br>- Inflammatory status (CRP / IL6).<br>- Nutritional indices used in heart failure<br>- Serum albumin.<br>- Overhydration (if reported).			
B. Are clear definitions / diagnostic criteria for important covariates provided?	Clear definitions / diagnostic cut offs are provided when defining covariates in the study.<br><br>In the case of reporting of comorbidity scores, the scoring indices used are clearly defined, along with the weighting individual comorbidities receive to calculate the total score.<br><br>In the case of diagnoses, the diagnostic criteria used to make that diagnosis are provided (i.e. how is diabetes defined).	There is an attempt to define / provide diagnostic criteria, but this is not clear in every instance.	The study makes no use of clear definitions / diagnostic cut offs when reporting covariate data in the study.	If no covariate data is provided, then this signalling item will be "unclear". If covariate data is reported, however, attempt should be made to clearly answer this question.
C. Are the definitions / measurements used to define covariates valid and reliable?	The methods used to measure the covariates are validated and published previously. In the case of serum / plasma assays, the assay serial numbers / protocols for deriving a value are clearly stated and whether the assay is serum / plasma is highlighted.	Some definitions are provided, but not to the level of granularity for a clear "yes" in this signalling item / question.	There is no confirmation in the text that the methods used to measure covariates are validated.**	It is unclear whether the methods used to measure covariates are validated; in such circumstances, it should be argued that the study is at high risk of biased estimates of effect with respect to the covariate measures**
D. Are the method and setting of covariate measurement the same in all participants?	Covariates are measured in the same way in all participants across the study.	N/A.	There are differences in the way covariates were measured between participants.	There is insufficient information presented in the study to assess whether there were systematic differences in covariate measurements between study participants.
E. Is the proportion of study participants with complete covariate data reported?	≥ 90% of study participants have complete covariate data within the study. Alternatively, in survival analyses, all covariates included (whether that be in univariable or multivariable analyses) have at least ≥ 90% data completeness.	< 90% of the study participants have complete covariate data, or alternatively within survival analyses, some covariates included have < 90% data completeness. However, justifications are provided for this (including justifiable and	< 90% of the study participants have complete covariate data, or alternatively within survival analyses, some covariates included have < 90% data completeness.	It is unclear what proportion of the study population have complete covariate data or the proportion of covariates within survival analyses have data completeness.

			valid attempts to address this: see signalling item below).		
	F. In the event of missing data, are imputation methods used, and if so, are they appropriately applied?	When imputation methods are used to handle missing data, the research question / methodology / nature of the data drives the method used (single vs multiple, random vs non-random methods). Furthermore, if completed case analysis is used, this is justified.	Imputation methods are used but only some details of the methods are stated. However, the method ultimately chosen appears appropriate.	Imputation methods are used to handle missing data but are based on untenable assumptions about data missingness mechanism. Furthermore, no specific justifications for the method applied are used.	It is unclear how missing data is handled.
	G. Are important covariates accounted for in the study design?	**The same criteria are used in 5.A. and therefore are not used for the classification of an optimal study.**			
	H. Are important covariates accounted for in the analysis?	If statistically appropriate, more than 5 core covariates are included, with ideally age, a measure of comorbidity and inflammation being included in the final multivariable survival analysis. Where this is not the case, the justification for this is clearly specified in the methods section.	Some core covariates are included in the final analysis, but age, a measure of comorbidity and inflammation are not all included.	Univariable / unadjusted analysis only reported.	It is unclear what covariates, if any, have been included in the analyses as it is not specified in the methods, the results section or in tabular form / figure legends / figures.
**6. Statistical Analysis and Reporting (SAR)**	**Optimal study – the statistical analysis is appropriate, with all primary and secondary outcomes being appropriately reported.**				
	A. Is there sufficient presentation of data to assess the adequacy of the analysis strategy used?	Covariates included within the final survival analyses have summary statistics presented within the results section, including adequate measures of dispersion. There are univariable statistical methods employed to assess measures of effect on the outcome variable. Authors do not rely solely on a single analysis to test the measure of effect but make use other analyses (such as Kaplan Meier plots and Log rank tests) to assess the consistency of the associations seen in the study. Model assumptions and goodness of fit are assessed.	There is reporting of covariate data (including summary statistics), but there may be incomplete or absent reporting of univariable measures of effect prior to final adjusted model reporting.	There is insufficient or inconsistent data presented in the study, such that statistical methods cannot be assessed for their adequacy in the study.	N/A.
	B. Is there sufficient detail presented in the methods or results section to understand the statistical modelling used and the conceptual	The methods for formulating the analysis plan are clear, including the approach taken to final multivariable model formulation (i.e directionality, conditioning, a-priori covariate entry).	There is some detail presented on how the final multivariable model were fitted, but not to the same degree as a clear "yes" to this signalling item.	There is no clear approach to building the final multivariable model.	N/A.

	framework for the analysis?				
	C. Is the selected statistical model appropriate for the design of the study?	Appropriate statistical methods are used to analyse the outcome measure (i.e. time to event data assessed using survival regression models). There is evidence of formal testing of the appropriateness of the method (i.e., Q-Q plots, Schoenfeld's residuals to assess proportional hazards assumption if relevant etc). There is no evidence of model overfitting (i.e. > 10 events per estimated regression parameter).	Appropriate statistical methods are used for data analysis, but formal testing of the appropriateness of the method are not reported / not performed. There is some evidence of model overfitting (e.g between 5 to 10 events per estimated regression parameter).	There is evidence that inappropriate statistical methods were used when handling outcome data, including evidence of overfitting the model.	Insufficient detail is provided to make an informed judgement with respect to the appropriateness of the statistical model.
	D. Is the association between the PFM and the outcome measure clearly described?	Appropriate measure of association described along with confidence intervals.	Appropriate measure of association is described, without confidence intervals.	Inappropriate measure of association used for selected statistical model.	N/A
	E. Can we be confident that there is no selective reporting of results?	We can be confident that there is no selective reporting of results within the study – i.e. full covariate measures of effect are provided for the final multivariable survival analysis, including those that are not associated with the outcome variable. A further signal for consistency / full reporting will be consistency of values between the text / the tables / figures.	Some aspects of a full "yes" response is reported, but you cannot be completely confident in your assessment of this signalling item.	There is clear evidence of selective reporting or contradictions in the reporting of the data within the study.	There is insufficient data presented to make an informed judgement with respect to selective data reporting.

Supplementary Figures

Supplementary Figure 1 – Risk of bias summary (using the QUIPS tool).

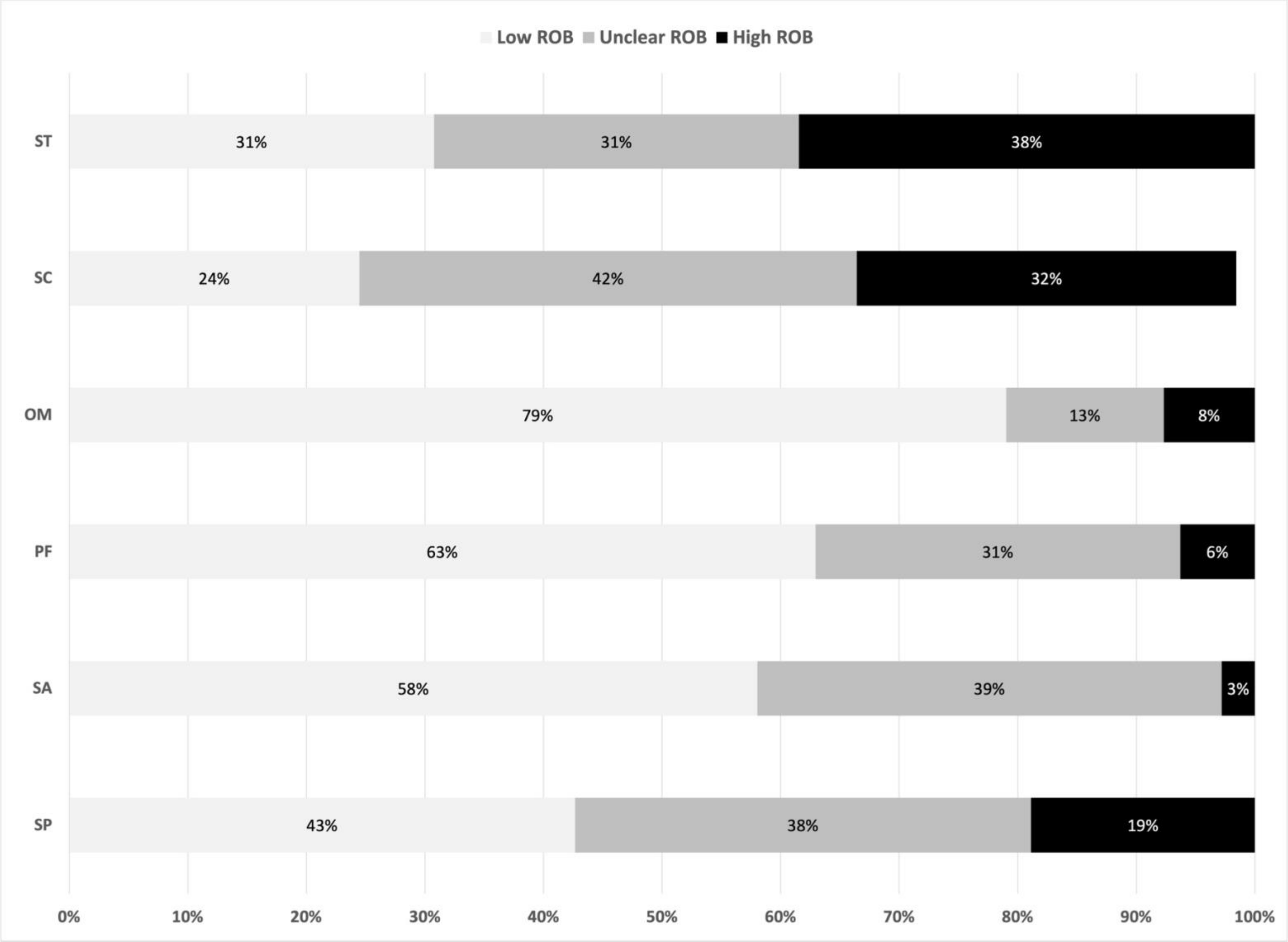

Box plot of proportion of studies rated as low, unclear or high risk of bias for each QUIPS domain. The QUIPS domains are abbreviated as follows: SP – study participation, SA – study attrition, PF – prognostic factor measurement, OM – outcome measurement, SC – study confounding and ST – statistical analyses and reporting.
